# Corneal stability comparison between prophylactic cross-linking with laser refractive surgery technique versus laser refractive surgery technique alone for myopia: a meta-analysis

**DOI:** 10.1007/s00417-025-06833-6

**Published:** 2025-09-11

**Authors:** Shuang-An Yang, Shiow-Wen Liou, Chun-Chen Chen

**Affiliations:** 1 Department of Epidemiology, Harvard TH Chan School of Public Health, Boston, MA USA; 2 Department of Ophthalmology, Taipei City Hospital, No.10, Sec. 4, Ren’ai Rd., Da’an District, Renai Branch, 106, Taipei City, Taiwan; 3 Department of Ophthalmology, Shin Kong Wu Ho-Su Memorial Hospital, Taipei, Taiwan; 4 Institute of Clinical Medicine, School of Medicine, National Yang Ming Chiao Tung University, Taipei, Taiwan

**Keywords:** Corneal cross-linking (CXL), Laser-assisted in situ keratomileusis (LASIK), Keratorefractive Lenticule Extraction (KLEx), Small Incision Lenticule Extraction (SMILE), Photorefractive Keratectomy (PRK), Myopia; Visual acuity (VA)

## Abstract

**Purpose:**

To summarize whether corneal cross-linking (CXL) with laser refractive surgery improves corneal stability more than laser surgery alone for myopia.

**Methods:**

Five electronic databases were systematically searched from inception to Aug, 2023. Studies comparing the effects of prophylactic corneal CXL with laser refractive surgery technique versus laser refractive surgery technique alone in myopic eyes were included. The included articles regarding prophylactic CXL combination with laser refractive surgery technique, riboflavin was all delivered into the stroma with similar accelerated protocols (irradiance of 30mW/cm^2^ for 60-90 seconds). Primary outcome measures included post-operative changes of keratometry, refraction, corneal thickness, endothelial cell density, and both corrected and uncorrected distance visual acuity (CDVA/UDVA) as the surrogate parameters to corneal stability, measured from post-operative 1-12 and 1-24 months. Meta-analysis was performed using random-effect models, and potential sources of heterogeneity were explored by subgroup analyses of surgery type and myopic level.

**Results:**

Twelve eligible studies with 1,252 eyes met our inclusion criteria. Compared to those receiving laser refractive surgery technique alone, patients who received prophylactic CXL with laser refractive surgery technique had significantly less UDVA and CDVA decrease within post-operative 12 months (UDVA:WMD -0.04, 95% CI -0.06-(-0.01), *p*=0.01/CDVA: WMD -0.01, 95% CI -0.03-(-0.00), *p*=0.02). Furthermore, patients with spherical equivalent $\le$ -5.0 Diopter (UDVA:WMD -0.04, 95% CI -0.07-(-0.02), *p*<0.001/ CDVA: WMD -0.02, 95% CI -0.03-(-0.01), *p*<0.001) or receiving LASIK procedures (UDVA:WMD -0.03, 95% CI -0.06-(-0.01), *p*=0.01) were associated with more profound visual stability after prophylactic CXL.

**Conclusion:**

For visual outcome, prophylactic CXL with laser refractive surgery, compared to laser refractive surgery alone, provided more stability within post-operative one-year among myopic patients with comparable efficacy and predictability. Particularly, those with higher myopia or receiving LASIK procedure as refractive surgery benefited more from prophylactic CXL.

**Supplementary Information:**

The online version contains supplementary material available at 10.1007/s00417-025-06833-6.

## Key messages

***What is known***

• Prophylactic cross-linking (CXL) combined with laser refractive surgery technique aimed at strengthening the cornea to prevent ectasia after refractive surgery, particularly in patients at risk for developing corneal instability.

***What is new***

• Prophylactic corneal CXL combined with laser surgery technique offers greater visual stability in the first year following surgery for myopic patients, compared to laser refractive surgery technique alone, while maintaining similar effectiveness and predictability.

• This beneficial effect is more substantial in highly myopic patients undergoing Laser Assisted in Situ Keratomileusis (LASIK) procedures.

**Supplementary Information:**

The online version contains supplementary material available at 10.1007/s00417-025-06833-6.

## Introduction

Myopia is the most common ocular disorder worldwide, affecting 33% of global population in 2020, with 4% having high myopia [1]. As myopic rates increase, more patients start to request corneal refractive surgery as an alternative to contact lenses or spectacles for permanent vision correction. Although corneal refractive surgery technique has become increasingly safe and predictable in past 20 years, post-refractive keratectasia remains a major concern,[2] which occurs when the tensile strength of cornea is significantly reduced, leading to inferior corneal steepening, myopic astigmatism worsening, and eventual loss of best corrected visual acuity [3]. Importantly, iatrogenic ectasia can occur not only in Laser-assisted in situ keratomileusis (LASIK) due to the corneal flap; but also in flap-less procedures like Photorefractive Keratectomy (PRK) and Keratorefractive Lenticule Extraction (KLEx), [4] especially in patients with high myopia and thin corneas [5]. Thus, in Asian countries with high myopic prevalence, post-refractive ectasia is not a rare complication, making prevention critically important.

Currently, treatment option for corneal ectasia is limited. Contact lenses is often difficult to fit, and many patients eventually require corneal transplantations [6]. In recent years, corneal crosslinking (CXL) has emerged as a promising new technique to halt ectasia progression [7, 8] by using riboflavin and ultraviolet A irradiation to strengthen the cornea [9, 10]. However, even CXL has shown the potential to partially reverse ectasia, only keratoplasty now could cure the disease once it occurred. Therefore, recent studies have instead focused on a paradigm shift while treating to prevent this serious complication, by combining CXL with the primary refractive surgery technique, so called “Xtra” procedure. However, the effectiveness of prophylactic CXL with refractive surgery technique in improving postoperative corneal stability remains controversial, necessitating a review of emerging evidence in myopic patients.

Therefore, the aim of this meta-analysis is to review the corneal stability of prophylactic CXL with refractive surgery technique compared to refractive surgery technique alone for myopia.

## Methods

This meta-analysis was conducted following the preferred reporting items for systemic reviews and meta-analyses (PRISMA) guidelines [11]. 

### Search strategy

Five electronic databases (PubMed, EMBASE, Web Of Science, Cochrane Central Register of Controlled Trials, and ClinicalTrial.gov) were searched from inception to 31, Aug 2023 to find all relevant studies without language restrictions. Using MESH term and free-text term, key words of “refractive surgery”, “laser-assisted in-situ keratomileusis”, “small incision lenticule extraction”, “photorefractive keratectomy”, “corneal cross-linking” and “myopia” were applied. Detailed database-specific search strategies are shown in the supplement (on-line resource 1). We also manually searched the reference lists of previous related meta-analysis and systemic reviews to identify additional relevant articles.

### Study inclusion and exclusion criteria

We included all randomized controlled trials (RCTs) and observational studies (prospective or retrospective cohorts) based on following criterias: (1) study population : patients with myopia (2) study intervention: CXL combined with laser refractive surgery technique versus laser refractive surgery technique alone (3) study outcomes: at least one of the main outcomes of interest — post-operative keratometry (K), manifest refraction spherical equivalent (MRSE), corneal thickness, endothelial cell density (ECD), uncorrected distance visual acuity (UDVA) or corrected distance visual acuity (CDVA) (4) study follow-up periods: for at least 12 months. Studies that recruited (1) patients with already known keratoconus or (2) patients with history of other ocular surface diseases (e.g., corneal dystrophy, herpetic eye) or (3) patients receiving sequential CXL after the laser refractive surgery technique were excluded from the analysis. Studies unpublished in peer-review journals or published in grey literature and conference abstracts were also excluded.

### Data extraction

S.A.Y and C.C.C independently reviewed all included studies and completed data extraction into the electronic database for following information: study characteristics (authors, study year, study design, study arm, follow-up length), patient and ocular characteristics including ectasia risk factors (number of patients and eyes, age, gender, baseline corneal biomechanical parameters and myopic level), intervention (type of laser refractive surgery technique), and main outcome measures of the study (post-operative parameters of interest). We specifically extracted data reported at one, three, six, twelve, and twenty-four months after the laser refractive surgery technique. If data at each specific follow-up period stated above (1. 3. 6. 12. 24 months) was unavailable, the outcome at distinct time point closet to those follow-up periods were used. Group consensus discussion was held to resolve any disagreement occurred between reviewers.

### Quality assessment

To appraise the study quality, risk of bias was assessed by the revised risk-of-bias tool (ROB- 2) in randomized trials [12, 13] and the Newcastle-Ottawa Scale (NOS) in non-randomized studies [14] for quality assessment. For RCTs, potential bias was assessed in five domains: randomization process, deviations from the intended interventions, missing outcome data, measurement of the outcome, and selection of the reported result, and categorized into three levels: ‘Low’, ‘Unclear’ or ‘High’. For observational studies, potential bias was evaluated using eight elements within three domains: Selection, Comparability, and Outcome. Each study was awarded a maximum of one star for each element in Selection and Outcome domain, and a maximum of two star for Comparability domain. Stars adding up to a score of seven to nine is represented as high quality, and a score of five to six is represented as adequate quality. Any discrepancies were resolved by group consensus discussion.

### Outcome measures

LASIK and KLEx are the major surgical procedures in the enrolled studies and visual recovery time in the LASIK or KLEx has been reported to be the first day and the second week respectively in the previous studies [15]. Regarding primary outcome measures, we specified mean post-operative changes of K, MRSE, corneal thickness, ECD, UDVA and CDVA as the surrogate parameters to corneal stability, measured from post-operative one-twelve, and one-twenty-four months. Secondary outcomes included (1) safety, defined as percentage of eyes with one or more lines of loss in CDVA (2) efficacy, defined as percentage of eyes achieving post-operative UDVA of 20/20, and 20/25 or better and (3) predictability, defined as defined as percentage of eyes achieving post-operative refraction within positive and negative 1 and 0.5 Diopter (D) of the target refraction at post-operative month twelve.

### Statistical analysis

Meta-analyses were performed using random-effect models for expected clinical heterogeneity. For continuous outcome measures, weighted mean difference (WMD) with 95% confidence intervals (CIs) was calculated as the treatment effect by Sidik-Jonkman method [16]. For dichotomous outcome measures, odds ratio (OR) and 95% CIs were estimated by Mantel-Haenszel method. Regarding the un-available standard deviation (SD) of change-between-follow-up-period measures, we imputed them from SDs at specific follow-up periods by using correlation coefficient according to the conversion formula outlined in Cochrane Handbook [17]. The details of these formulas were described in the supplement (on-line resource 2).

Heterogeneity across the studies was assessed by *I*^2^ and Q statistics [17]. If high heterogeneity (*I*^2^ > 50%) was revealed, potential sources of heterogeneity were further explored by subgroup analysis. Subgroup analyses were stratified by (1) type of refractive surgery technique and (2) myopic level, with a threshold of ≤ − 5.00 D representing high myopia by World Health Organization [18]. Meta-regression was performed to accommodate for baseline ectasia risk heterogeneity. To assess the robustness of the results, sensitivity analyses were conducted to compare the pooled effect estimates by (1) different correlation coefficients of 0.5, 0.6, 0.7, 0.8, which are commonly used in previous meta-analysis in the medical research [19, 20] (2) fixed-effect versus random-effect models [21] and (3) outlier removal. Potential publication bias was evaluated by funnel plots visually and egger’s test statistically [22]. A probability of <= 0.05 was considered as statistical significance. All statistical analyses were performed using STATA software (Stata MP 16.0).

## Results

### Literature search and selection

**Fig. 1:**
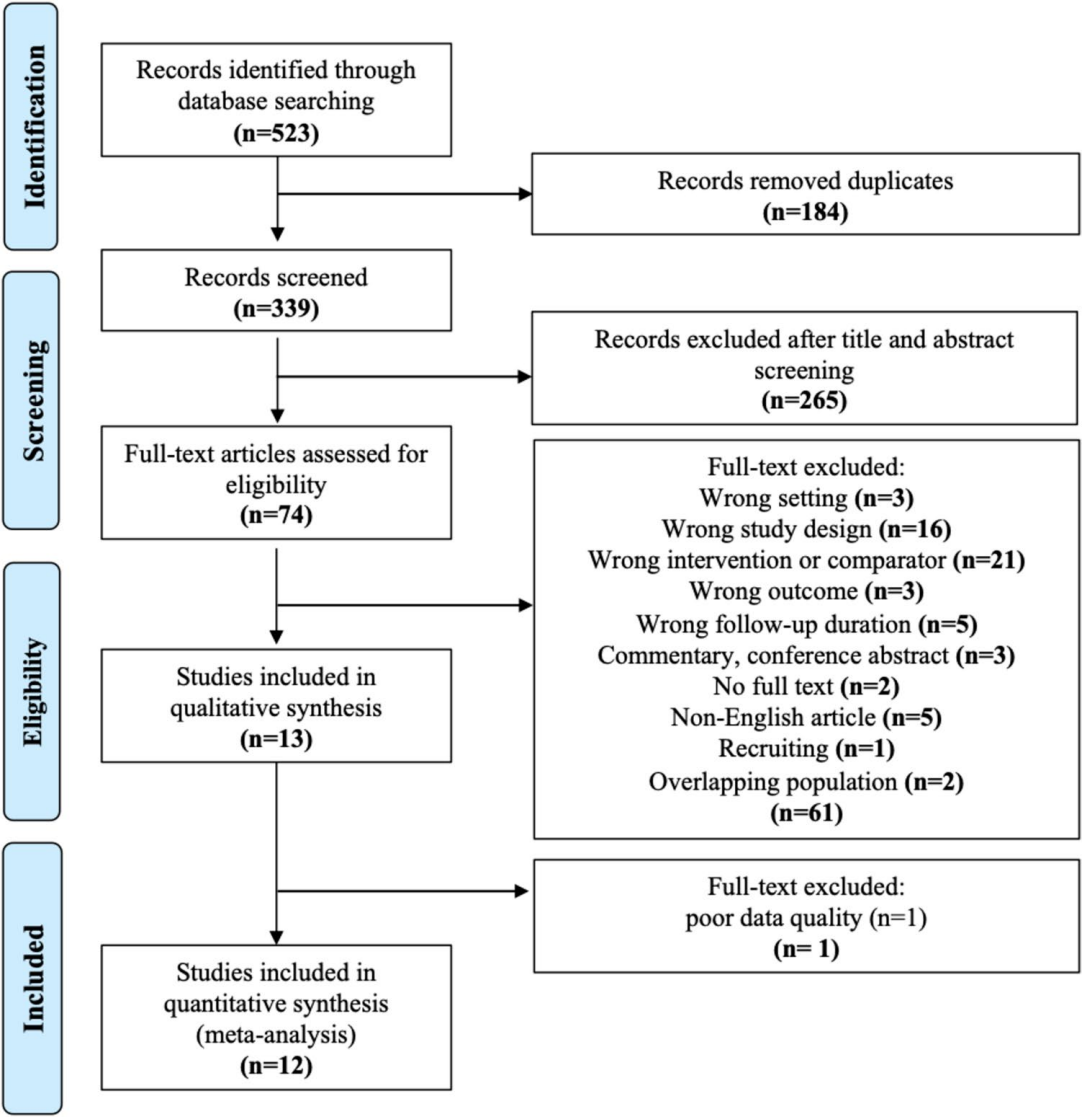
PRISMA flow diagram. Legend: None

Overall, 523 potentially relevant studies were identified from initial literature search, with 339 studies remained after removing duplicates. Of these, 74 studies remained eligible after title and abstract screening and 61 studies were excluded on full-text screening. After then, one study was removed during the assessment of data quality given small sample size. Finally, 12 studies passed the qualitative assessment and were enrolled in our meta-analysis, including three RCTs, [23–25] one prospective, [26] and eight retrospective studies (Figure 1) [27–34].

### Eligible study characteristics

The baseline characteristics of twelve studies were summarized in Table 1. Regarding type of refractive surgery techniques, LASIK, SMILE, and PRK procedures were explored by six studies [23–26, 28, 35], four studies [29–31, 34], and two studies [32, 33], respectively.

**Table1:** Characteristics of the 12 selected studies

Author	Year	Study design	Surgery Technique	Total Eye No.	Total Pt No.	follow-up(mth)	Delivery of Riboflavin	Riboflavin concentration (%)/treatment time(s)	Radiation power (J/cm2)	Age, year (mean±SD)	Preop MRSE (D)	Preop Cyl (D)	RST (mm)	Preop CT (mm) *Thinnest	Preop ECD (/mm2)
LASIK															
Dong et al.	2022	RCT	LASIK+CXL LASIK	25 25	25	24	Epi-off ACXL	0.22/90	2.7 J/cm2 (30 mW/cm2, 90 s)	27.7 ± 7 27.7 ± 7	− 8.76± 1.52 − 8.64± 1.50	− 1.17± 0.85 − 1.14± 0.75	N/R	549 ± 28* 548 ± 26*	3023.3 ± 277.8 3009.3 ± 212
Kohnen et al.	2020	RCT	LASIK+CXL LASIK	26 26	52 26	12	Epi-off ACXL	0.25/90	2.7 J/cm2 (30 mW/cm2, 90 s)	35 ± 13 35 ± 13	− 7.5± 1.12 − 7.35± 1.15	− 0.66± 0.63 − 0.68± 0.6	N/R	554 ± 27 554 ± 27	2616 ± 196 2647 ± 226
Kanellopoulos et al.	2015	RCT	LASIK+CXL LASIK	65 75	65 75	24	Epi-off ACXL	0.1/60	2.4 J (30 mW/cm2, 80 s)	27.5 ± 6.1 24.2 ± 5.8	− 6.67± 2.14 − 5.44± 1.99	N/R	329.22 ± 32.15 344.34 ± 20.55	545.96 ± 33.93 553.51 ± 19.11	N/R
Seiler et al.	2015	Prospective study	LASIK+CXL LASIK	76 76	76 76	12	Epi-off ACXL	0.5/120	2.7 J (9 mW/cm2, 300 s)	31.6 ± 8.5 33.5 ± 7.9	− 5.3± 2.9 − 4.9± 1.9	− 0.9± 0.7 − 0.7± 0.6	365 ± 50	545 ± 41 ±	N/R
Zhang et al.	2022	Retrospective study	FS-LASIK+CXL FS-LASIK	94 86	49 50	12	Epi-off ACXL	0.22/120	1.8–2.6 J (30 mW/cm2, 60 - 87 s)	22.1 ± 5 23.5 ± 6.7	− 5.29± 2.02 − 5.31± 1.97	− 0.78± 0.69 − 0.75± 0.56	366.72 ± 29.69 355.14 ± 32.32	543.8 ± 27 538.0 ± 23.0	3005 ± 74.88
Tomita et al.	2014	Retrospective study	LASIK+CXL LASIK	24 24	24 24	12	Epi-off ACXL	0.1/60	1.8 J (30 mW/cm2, 60 s)	30.4 ± 4.7 30.4 ± 4.7	− 4.45± 2.18 − 4.43± 2.21	− 0.83± 0.85 − 0.76± 0.80	N/R	N/R	3011.3 ± 281.3 3020 ± 290/7
SMILE															
Brar et al.	2022	Retrospective study	SMILE+CXL SMILE	54 54	27 27	21.81 22.18	Epi-off ACXL	0.22/60	3.4 J (45 mW/cm2, 75 s)	25.85 ± 4.06 25.96 ± 2.71	− 4.04± 1.94 − 4.24± 1.84	− 0.95± 1.06 − 0.71± 0.68	303.75 ± 30.65 314.13 ± 31.30	515.29 ± 26.45 527.88 ± 29.47	N/R
Chabib et al.	2022	Retrospective study	SMILE+CXL SMILE	30 40	15 20	24	Epi-off ACXL	0.22/90	2.7 J (30 mW/cm2, 90 s)	33.20 ± 7.98 36.45 ± 8.84	− 6.27± 2.95 − 7.18± 1.21	N/R	N/R	523.4 ± 37.01* 543.9 ± 22.85*	N/R
Liu et al.	2021	Retrospective study	SMILE+CXL SMILE	36 40	36 40	12	Epi-off ACXL	0.25/90	2.7 J (30 mW/cm2, 90 s)	21.64 ± 4.12 22.30 ± 4.87	− 6.13± 1.90 − 6.23±.1.46	− 1.31± 0.72 − 1.43± 0.73	288.11 ± 29.26 328.87 ± 42.46	528.66 ± 28.09 549.81 ± 32.79	N/R
Osman et al.	2019	Retrospective study	SMILE+CXL SMILE	30 30	15 15	12	Epi-off ACXL	0.1/900	3.2 J (18 mW/cm2, 180 s)	24.3 ± 5.9 25.2 ± 5.1	− 8.6± 1.1 − 8.2± 1.2	N/R	N/R	495.1 ± 22.6 498.4 ± 21.1	2816 ± 288 2840 ± 278
PRK															
Sachdev et al.	2018	Retrospective study	PRK+CXL PRK	109 118	52 56	12	Epi-off ACXL	0.25/90	2.7 J (30 mW/cm2, 90 s)	24.19 ± 3.54 25.92 ± 4.91	− 3.64± 1.44 − 3.38± 1.65	− 0.89± 0.65 − 1.05± 0.94	N/R	477.7 ± 19.31 479.7 ± 29.37	2514.3 ± 180.7 2602 ± 201.1
Lee et al.	2017	Retrospective study	tRPK+CXL tPRK	47 42	47 42	12	Epi-off ACXL	0.1/120	2.7 J (30 mW/cm2, 90 s)	27.3 ± 4.7 28.0 ± 4.7	− 6.22± 2.74 − 5.63± 1.53	− 1.64± 1.13 − 1.31± 1.14	N/R	543.1 ± 22.0 551.3 ± 25.8	3070 ± 303 3092 ± 168

*CXL* corneal cross-linking, *ACXL* accelerated cross-linking, *LASIK*, Lenticule Extraction with Collagen Cross-linking, *SMILE* Small Incision Lenticule Extraction, *PRK* Photorefractive Keratectomy, *UDVA* Uncorrected visual acuity, *CDVA* corrected distant visual acuity, *I* intervention group, *C* control group, *Pre-op* pre-operative, *MRSE* Manifest Refraction Spherical Equivalent, *Cyl* Cylinder, *RST* residual stromal thickness, *CT* corneal thickness, *ECD* endothelial cell density, *N/R* not reported

A total of 858 patients with 1,252 eyes received one of the refractive surgery techniques above were included, with 466 patients (622 eyes) in the LASIK group, 195 patients (314 eyes) in the SMILE group, and 197 patients (316 eyes) in the PRK group. Baseline characteristics associated with ectasia risks were also shown in Table1. The median age of all study participants was 27.77 (IQR:24.92–31.48) years old. Mean proportion of male was 40.38 ± 12.64%. The mean baseline MRSE was − 6.03 ± 1.60 D, cylinder refraction was 0.98 ± 0.27 D, central corneal thickness was 528.91. ± 27.61 mm, and ECD was 2856.04 ± 219.94 cells/mm^2^ respectively. The median follow-up duration for twelve included studies was 12 (IQR: 12–18) months.

## Risk of bias assessment

### Randomized controlled trials

The quality of three RCTs was generally high, with few “some concerns” risk of bias (online resource 3) [23–25]. “Some concerns” risk of bias was raised in the domain of allocation sequence concealment and measurement of the outcome.

### Observational studies

Nine studies were qualified for adequate quality (online resource 4) [26, 28, 29, 31–36]. Risk of bias were considered in the Comparability and Outcome domain because adjustment was not performed and the follow-up rate information was missed in certain studies. However, most studies had declaration of follow-up completeness that provide the reliable outcome assessment.

## Primary outcome – corneal stability

### UDVA/CDVA stability

#### Overall analysis

**Fig. 2:**
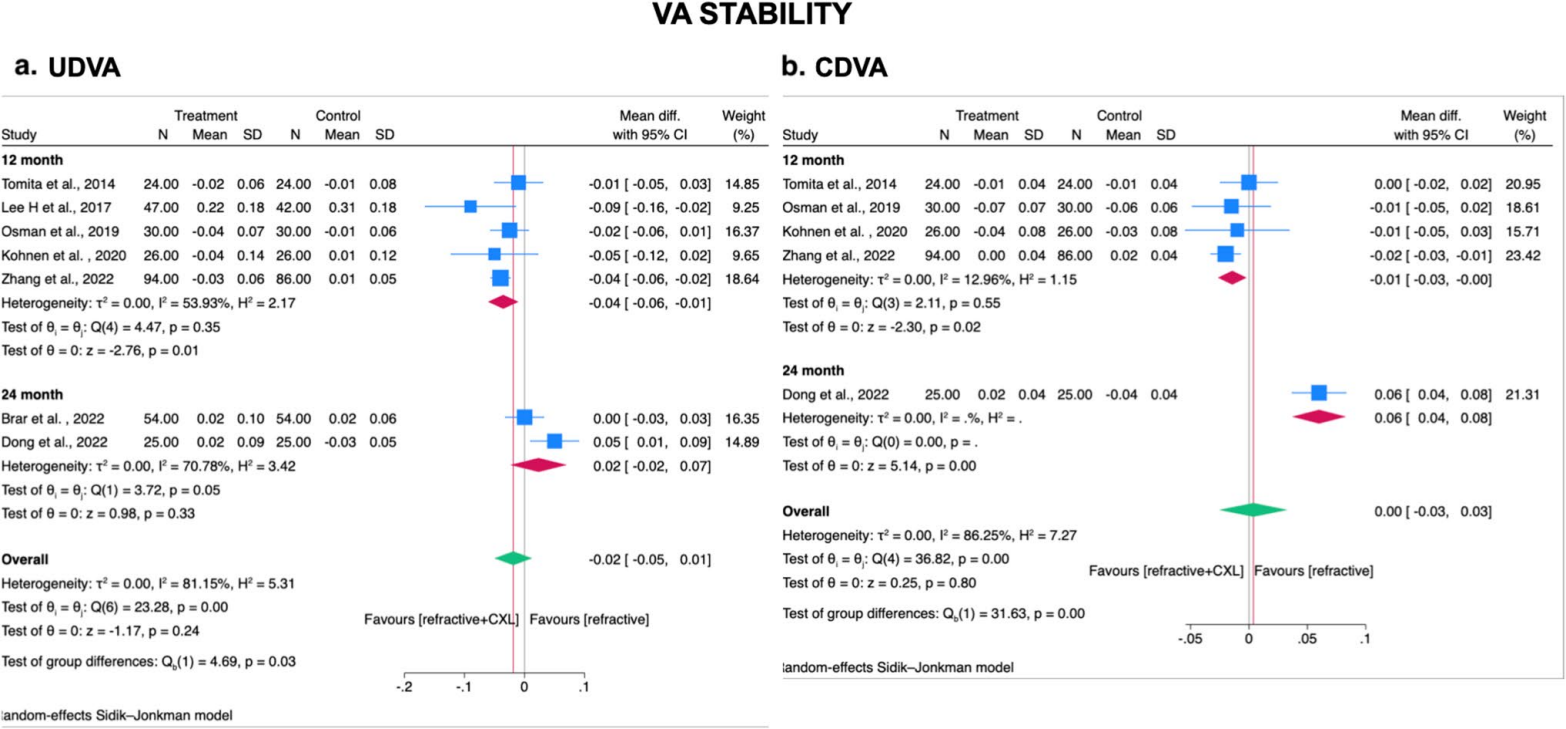
Forest plot for comparison of prophylactic CXL plus laser refractive surgery technique with laser refractive surgery technique alone on stability of visual acuity in myopic patients at 1–12 and 1–24 months. CXL, cross-linking; UDVA, uncorrected distance visual acuity; CDVA, corrected distance visual acuity; SD, standard deviations. Legend: The size of squares is proportional to the weight of each study. Horizontal lines indicate the 95% confidence intervals (CI) of mean difference estimate in each study; diamonds, the pooled estimate with 95% CI; N, the number of eyes at baseline; and SD, standard deviations

Total seven studies (2 RCT and 5 observational studies) [23, 25, 28, 29, 32, 34, 35] reported both UDVA and CDVA post-operatively. Among them, five studies (1 RCT and 4 observational studies) [23, 28, 29, 32, 35] reported visual outcome within post-operative 12 months and two studies (1 RCT and 1 observational study) [25, 34] reported visual outcome at post-operative 24 months. The overall meta-analysis indicated that patients who received prophylactic CXL with laser refractive surgery technique, on average, had significantly less UDVA and CDVA loss from postoperative from 1–12 months than those receiving laser refractive surgery technique alone (UDVA WMD = − 0.04 logMAR; 95% CI, − 0.06 to − 0.01 logMAR; *I*^2^ = 53.93%; *p*= 0.01; Figure 2a /CDVA WMD = − 0.01 logMAR; 95% CI, − 0.03 to − 0.00 logMAR; *I*^2^ = 12.96%; *p* = 0.02; Figure 2b); while no significant difference in visual stability was noted between laser refractive surgery technique with and without prophylactic CXL within post-operative 1–24 month (Figure 2).

We additionally performed a sensitivity analysis by removing the study by Dong et al. as the outlier given the confidential interval of the study doesn’t overlap with the overall confidential interval, which produced a more significantly less UDVA and CDVA loss in prophylactic CXL with laser refractive surgery technique than those receiving laser refractive surgery technique alone (online resource 5).

#### Subgroup analysis-study design

Subgroup analysis by study design revealed that in observational studies there was a significant less UDVA and CDVA loss in prophylactic CXL with laser refractive surgery technique group (UDVA WMD= − 0.03; 95% CI, − 0.05 to 0.00; *I*^2^ = 71.49) (CDVA WMD = − 0.01; 95% CI, − 0.03 to − 0.00; *I*^2^ = 24.77); while in RCTs there was on average a more UDVA and CDVA loss comparing prophylactic CXL with laser refractive surgery technique versus laser refractive surgery technique alone but lack of statistical significance (UDVA WMD = 0.01; 95% CI, − 0.09 to 0.10; *I*^2^ = 80.8)(CDVA WMD = 0.03; 95% CI, − 0.04 to 0.09; *I*^2^ = 85.7). The results exhibited no significant heterogeneity between subgroups (UDVA *p*= 0.52, and CDVA *p*= 0.22) (online resource 6).

#### Subgroup analysis-Myopic level (1–12 month)

**Fig. 3:**
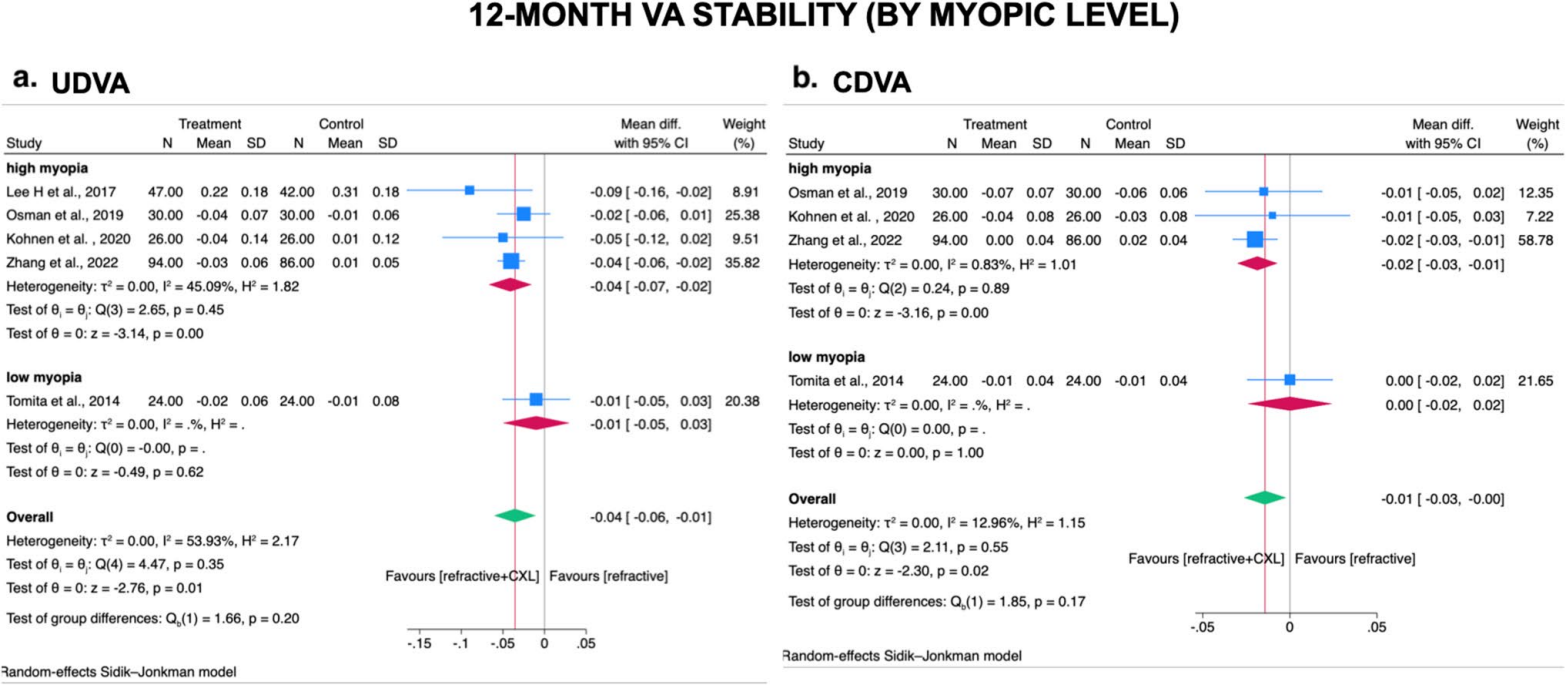
Forest plot after stratification by myopic level (MRSE <= − 5.0D; MRSE > − 5.0D) for comparison of prophylactic CXL plus laser refractive surgery technique with laser refractive surgery technique alone on stability of **A** UDVA and **B** CDVA at 1–12 and 1–24 months. CXL, cross-linking; MRSE, manifest refraction spherical equivalent; UDVA, uncorrected distance visual acuity; CDVA, corrected distance visual acuity; SD, standard deviations. Legend: The size of squares is proportional to the weight of each study. Horizontal lines indicate the 95% confidence intervals (CI) of mean difference estimate in each study; diamonds, the pooled estimate with 95% CI; N, the number of eyes at baseline; and SD, standard deviations.

Stratified by myopic level, four studies recruited patients with high myopia (SE ≤ − 5.00 D), [23, 29, 32, 35] and one study recruited patients with low myopia (SE $\ge$− 5.00 D) [28]. Subgroup analysis showed that patients with high myopia experienced a significantly less UDVA and CDVA decline after CXL plus laser refractive surgery technique than after laser surgery technique alone from post-operative 1–12 months (UDVA WMD = − 0.04 logMAR; 95% CI, − 0.07 to − 0.02 logMAR; *I*^2^ = 45.09%; Fig. 3A/CDVA WMD = − 0.02 logMAR; 95% CI, − 0.03 to − 0.01 logMAR; *I*^2^ = 0.83%; Fig. 3B). However, patients with low myopia seems to have no significant difference in both UDVA and CDVA change either after laser refractive surgery technique with or without prophylactic CXL (UDVA WMD = − 0.01 logMAR; 95% CI, − 0.05 to 0.03 logMAR; Fig. 3A /CDVA WMD = 0.00 logMAR; 95% CI, − 0.02 to + 0.02 logMAR; Fig. 3B). Subgroup difference was insignificant in post-operative 1–12 months (*p* = 0.20 and *p* = 0.17, respectively).

#### Subgroup analysis-Refractive surgery technique (1–12 month)

**Fig. 4:**
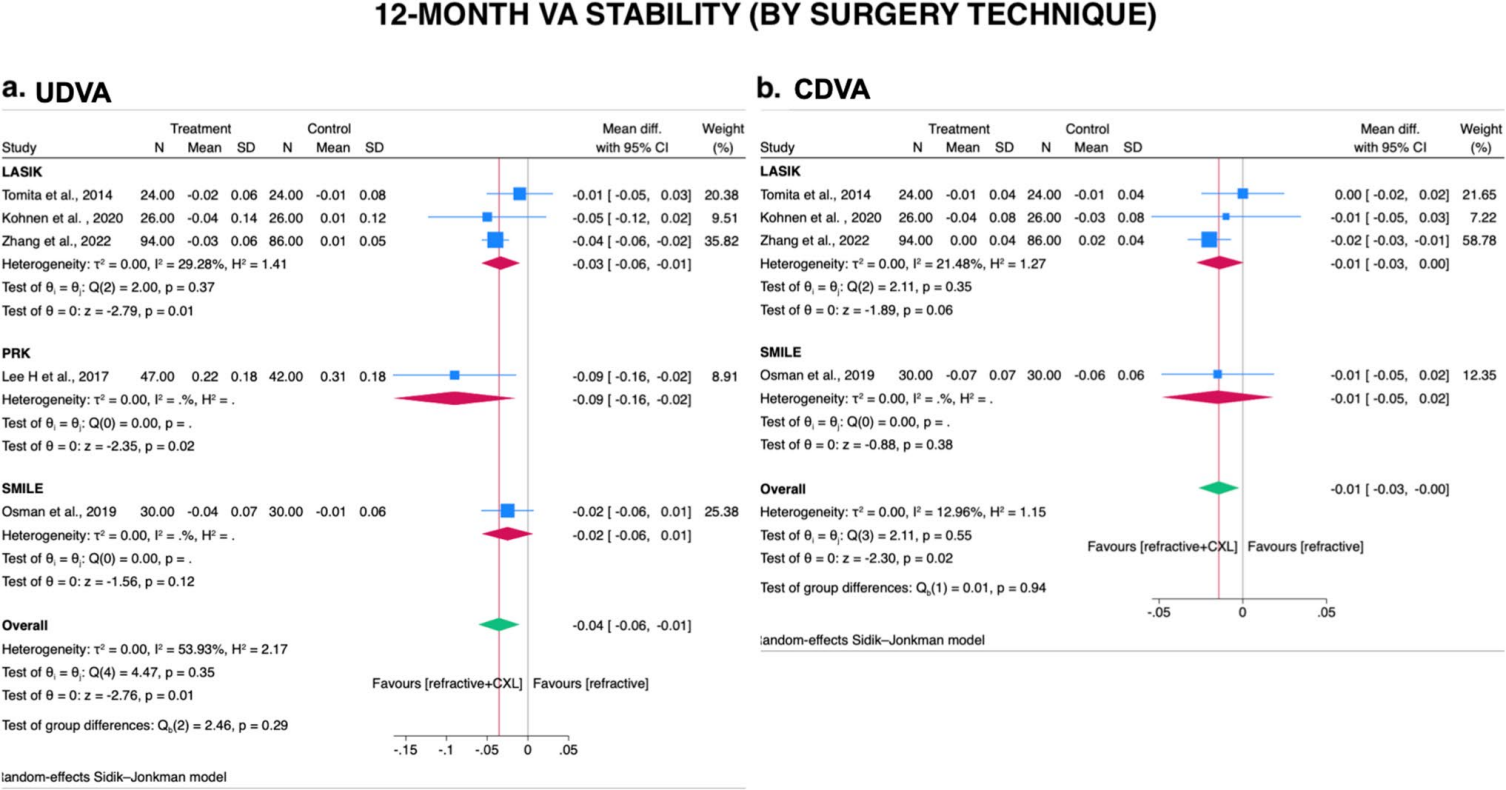
Forest plot after stratification by laser refractive surgery technique (LASIK.SMILE.PRK) for comparison of prophylactic CXL plus laser refractive surgery technique with laser refractive surgery technique alone on stability of **A** UDVA and **B** CDVA at 1–12 and 1–24 months. CXL, cross-linking; UDVA, uncorrected distance visual acuity; CDVA, corrected distance visual acuity; SD, standard deviations. Legend: The size of squares is proportional to the weight of each study. Horizontal lines indicate the 95% confidence intervals (CI) of mean difference estimate in each study; diamonds, the pooled estimate with 95% CI; N, the number of eyes at baseline; and SD, standard deviations

Stratified by refractive surgery techniques, three studies evaluating LASIK [23, 28, 35], one study evaluating SMILE [29], and one evaluating PRK [32]. Subgroup analysis revealed that CXL plus LASIK procedure, compared to LASIK procedure alone, provided significantly less UDVA decline in post-operative 1–12 months (UDVA WMD = − 0.03 logMAR; 95% CI, − 0.06 to − 0.01 logMAR; *I*^2^ = 29.28 %; Fig. 4A). However, SMILE and PRK procedures with or without prophylactic CXL provided similar VA decline during follow-up periods (UDVA WMD = − 0.02 logMAR; 95% CI, − 0.06 to 0.01 logMAR; Fig. 4A). No significant heterogeneity between subgroups were exhibited (UDVA/CDVA, *p*= 0.29/*p*= 0.94).

### K stability

Five studies (2 RCT and 3 observational studies) [25, 28–30, 34] provided data regarding K change, with three studies reporting data from 1–12 months and two from 1–24 months. No significant difference were found in K steeping between CXL plus laser refractive surgery technique group and laser refractive surgery technique alone group from both post-operative 1–12 months and 1 - 24 months, with low heterogeneity (1–12 months: WMD = − 0.26; 95% CI, − 0.83 to 0.31; *I*^2^ = 5.85 %; *p*= 0.37/1–24 months: WMD = 0.20; 95% CI, − 0.30 to 0.71; *I*^2^ = 0.37%; *p*= 0.43; online resource 7).

### MRSE stability

Nine studies (2 RCT and 7 observational studies) [23, 25, 28–32, 34, 35] reported MRSE at post-operative 12-month and 24-month. Meta-analysis showed no significant difference between CXL plus laser refractive surgery technique group and laser refractive surgery technique alone group, no matter from post-operative 1–12 months or 1–24 months (1–12 months: WMD = 0.03 D; 95% CI, − 0.02 to 0.08 D; *I*^2^ = 19.80%; *p*= 0.25/1–24 months: WMD = 0.09 D; 95% CI, − 0.03 to 0.21 D; *I*^2^ = 37.37%; *p*= 0.16; online resource 8).

### Corneal thickness stability

Two studies (1 RCTs and 1 observational studies) [23, 29] presented detailed data of corneal thickness at post-operative 12-month. Meta-analysis showed no significant difference in corneal thickness change between two groups from post-operative 1–12 months, with low heterogeneity (1–12 months: WMD = 0.07 mm; 95% CI, − 11.15 to 11.30 mm; *I*^2^ = 0.00%; *p*= 0.99; online resource 9).

### ECD stability

Four studies (2 RCTs and 2 observational studies) [23, 25, 28, 29] reported ECD at post-operative 12-month and 24-month. In the meta-analysis, no significant difference in ECD decrease were found between two groups from 1–12 and 1–24 months post-operatively (1–12 months: WMD = − 2.16 cells/mm^2^; 95% CI, − 66.14 to 61.81 cells/mm^2^; *I*^2^ = 0.00%; *p*= 0.95/1–24 months: WMD = 26.60 cells/mm^2^; 95% CI, − 86.28 to 139.48 cells/mm^2^; *p*= 0.64; online resource: 10).

### Secondary outcome – Efficacy

**Fig. 5:**
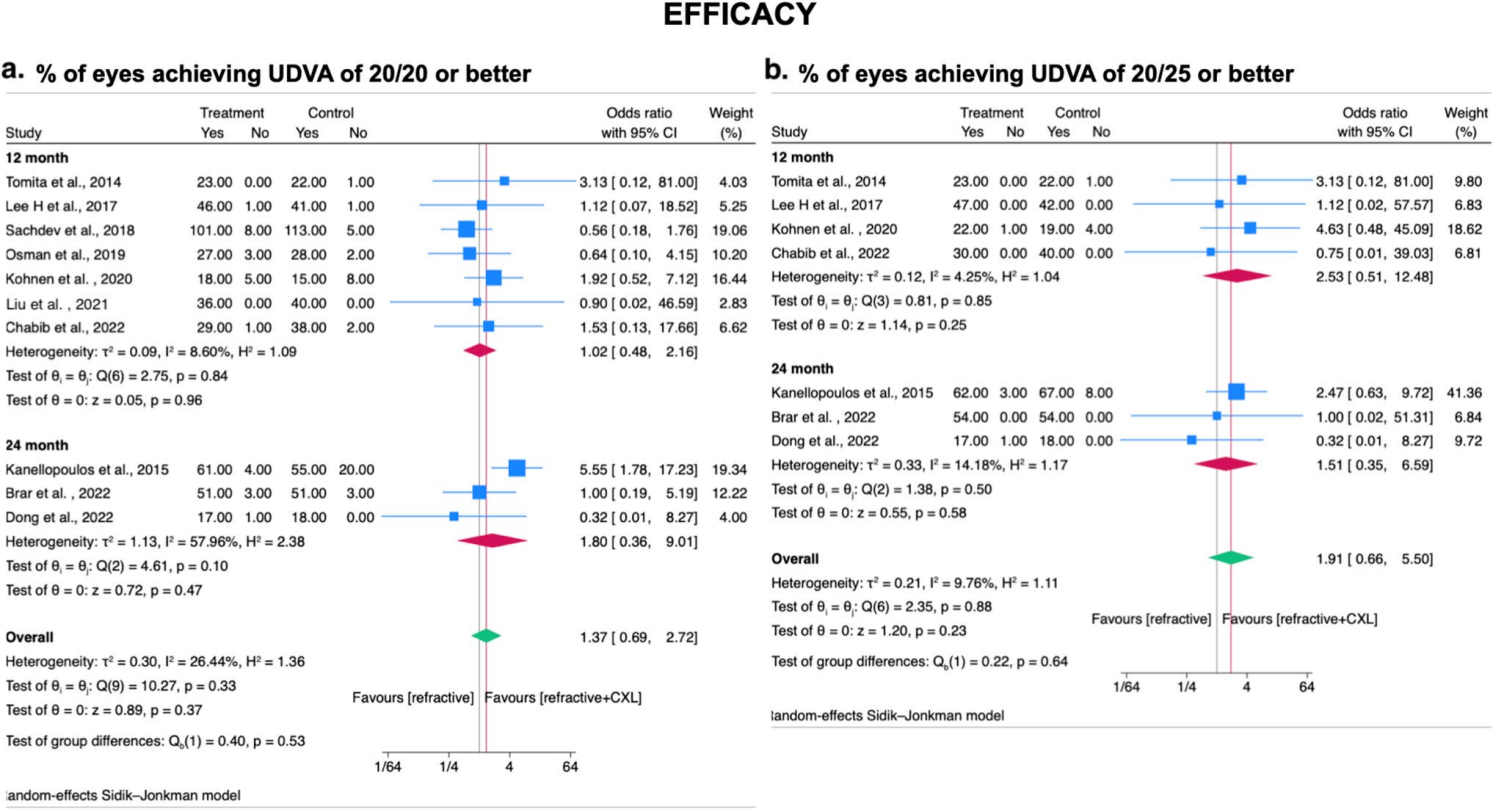
Forest plot for comparison of prophylactic CXL plus laser refractive surgery technique with laser refractive surgery technique alone on efficacy in myopic patients at post-operative month twelve and month twenty-four. Efficacy defined as **A** % of eyes achieving of UDVA 20/20 or better **B** % of eyes achieving of UDVA 20/25 or better. CXL, cross-linking; SD, standard deviations. Legend: The size of squares is proportional to the weight of each study. Horizontal lines indicate the 95% confidence intervals (CI) of mean difference estimate in each study; diamonds, the pooled estimate with 95% CI; N, the number of eyes at baseline; and SD, standard deviation

In terms of efficacy in our meta-analysis (3 RCTs and 7 observational studies), [23–25, 28–34] no significant difference in proportion of eyes achieving UDVA of 20/20 or 20/25 between CXL plus laser refractive surgery technique group and laser refractive surgery technique alone group at postoperative month twelve and month twenty-four (UDVA 20/20: OR= 1.37; 95% CI, 0.69–2.72; *I*^2^ = 26.44%; *p* = 0.37; Fig. 5A) (UDVA 20/25: OR= 1.91; 95% CI, 0.66–5.50; *I*^2^ = 9.76%; *p* = 0.23; Fig. 5B).

### Secondary outcome – Predictability

**Fig. 6:**
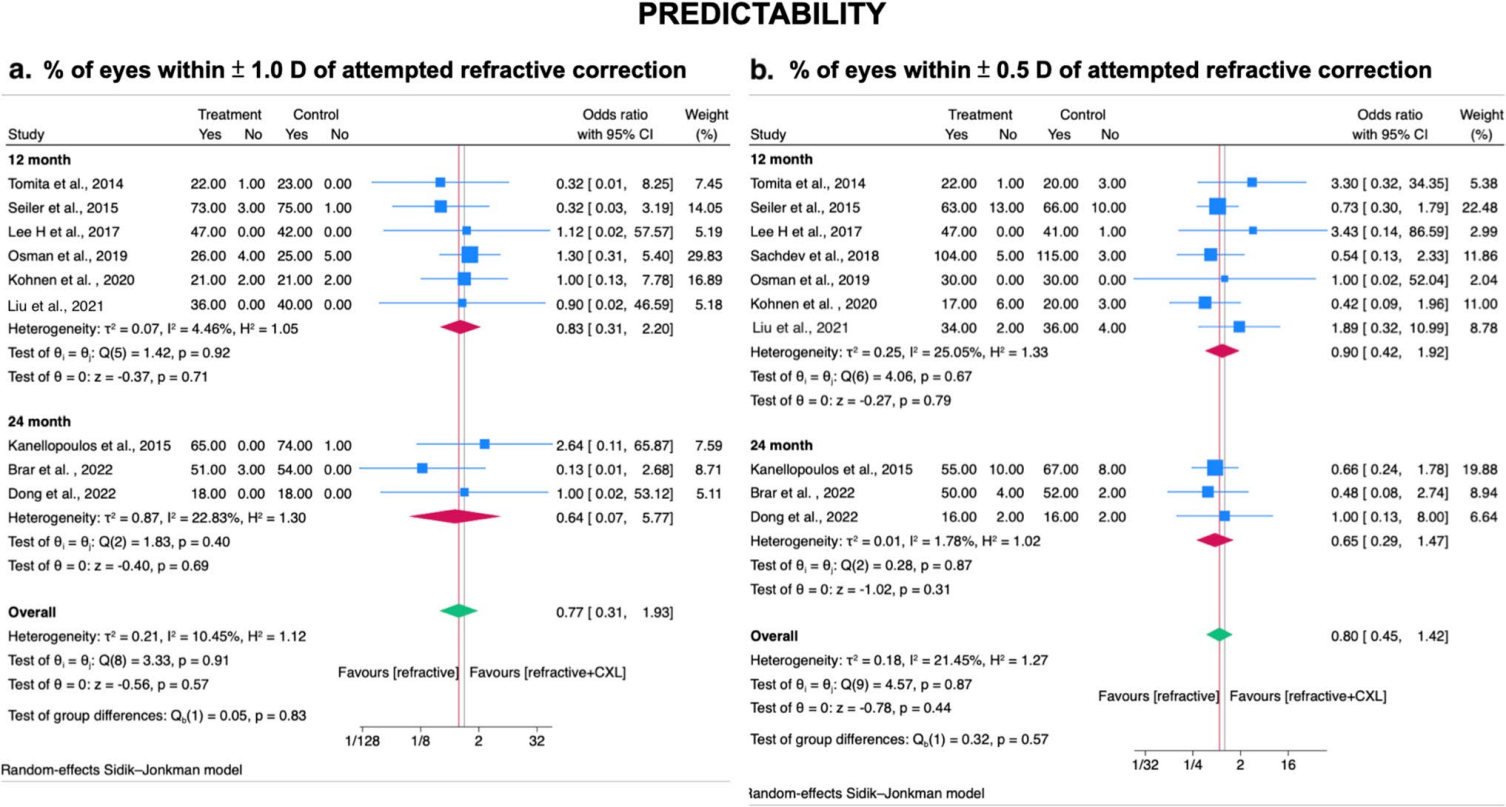
Forest plot for comparison of prophylactic CXL plus laser refractive surgery technique with laser refractive surgery technique alone on predictability in myopic patients at post-operative month twelve and month twenty- four. Predictability defined as **A** % of eyes achieving of eyes within ± 1.0 D of attempted refraction correction **B** % of eyes achieving within ± 0.5 D of attempted refraction correction. CXL, cross-linking; SD, standard deviations. Legend: The size of squares is proportional to the weight of each study. Horizontal lines indicate the 95% confidence intervals (CI) of mean difference estimate in each study; diamonds, the pooled estimate with 95% CI; N, the number of eyes at baseline; and SD, standard deviation

Regarding predictability, overall meta-analysis from ten studies (3 RCTs and 7 observational studies) showed no significant difference between two groups in the proportion of eyes within positive and negative 1 or 0.5 D of the target refraction at postoperative month twelve and month twenty- four (± 1.0 D: OR= 0.77; 95% CI, 0.31–1.93; *I*^2^ = 10.45%; *p* = 0.57; Fig. 6A) (± 0.5 D: OR= 0.80; 95% CI, 0.45–1.42; *I*^2^ = 21.45%; *p* = 0.44; Fig. 6B).

### Secondary outcome – Safety

**Fig. 7:**
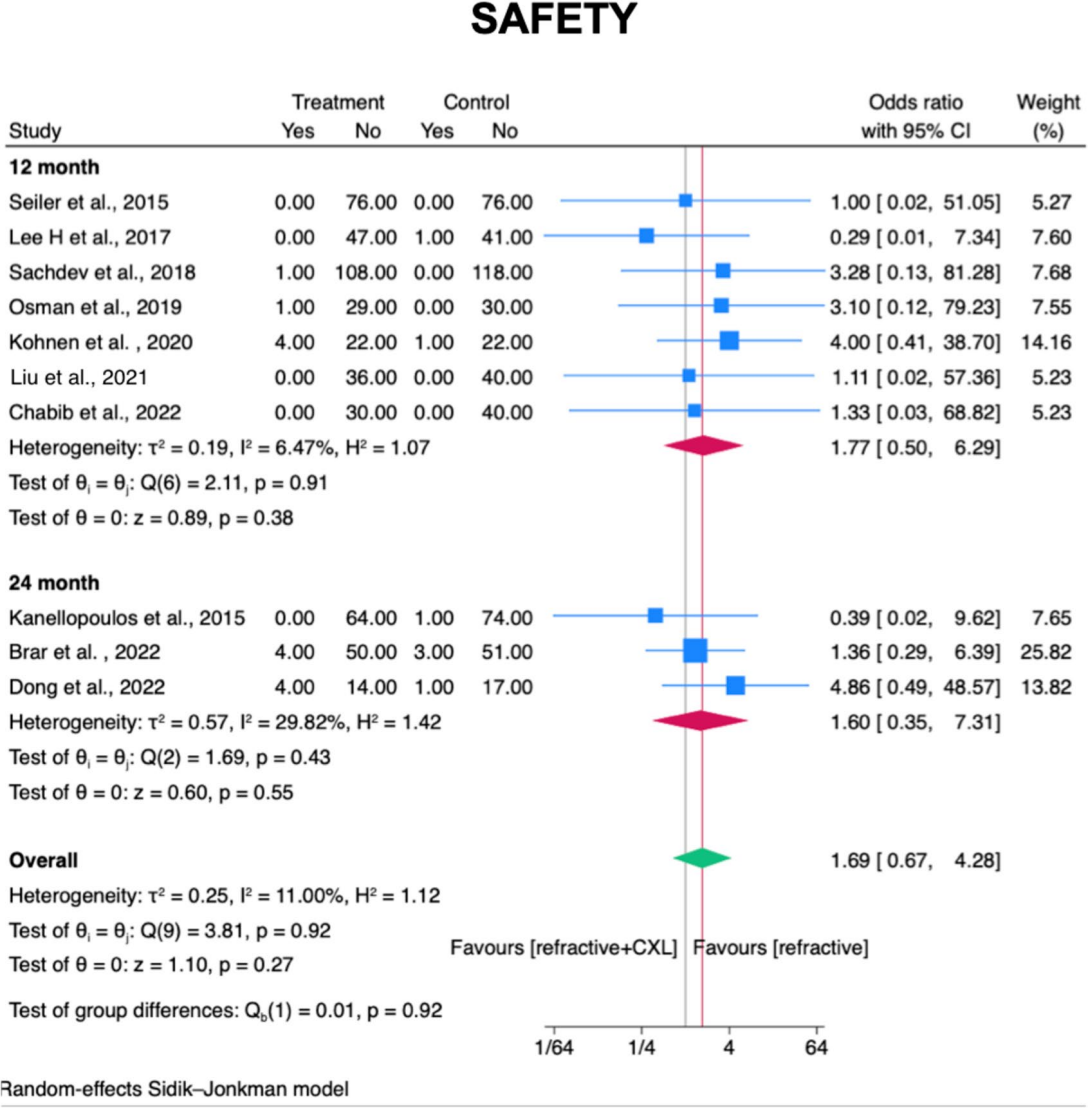
Forest plot for comparison of prophylactic CXL plus laser refractive surgery technique with laser refractive surgery technique alone on safety in myopic patients at post-operative month twelve and month twenty-four. Safety defined as % of eyes with one or more lines of loss in CDVA. CXL, cross-linking; SD, standard deviations. Legend: The size of squares is proportional to the weight of each study. Horizontal lines indicate the 95% confidence intervals (CI) of mean difference estimate in each study; diamonds, the pooled estimate with 95% CI; N, the number of eyes at baseline; and SD, standard deviation

Among ten studies reporting (3 RCTs and 7 observational studies) [23–26, 29–34] safety outcome, there is no significant difference in the proportion of eyes that CDVA loss of one or more in Snellen lines found between laser surgery technique with and without prophylactic CXL group at 12 months and 24 months post-operatively (OR = 1.69; 95% CI, 0.67–4.28; *I*^2^ = 11.00%; *p*= 0.27; Fig. 7), no matter by myopic level or by laser refractive surgery technique type (online resource 11).

### Sensitivity analysis

We repeated the meta-analysis using different correlation coefficient values (0.5, 0.6, 0.7, and 0.8) to impute the SDs of the mean UDVA/CDVA//K/corneal thickness/ECD changes and produced consistent outcome measures (Online resource 12).

### Publication bias

Overall, mild asymmetric funnel plot for the primary outcome of corneal stability were examined by visual inspection (online resource 13). More negative mean difference of UDVA/CDVA stability than positive mean difference of UDVA/CDVA indicates the presence of the heterogeneity between included studies and less methodological rigor in smaller studies. However, there was no significant small-study effect found by egger’s test (*p* > 0.05).

## Discussion

### Summary of the main findings

Despite its rarity, cornea ectasia is the most adverse complication in corneal refractive surgery that must be prevented to preserve visual potential. Corneal CXL, by strengthening collagen fibers, is currently the only intervention to halt the progression of post-refractive ectasia but there is still no consensus regarding the role of prophylactic CXL with refractive surgery technique. This meta-analysis concluded that prophylactic CXL with laser refractive surgery technique provides more stable visual outcomes than laser surgery technique alone one-year post-operatively with comparable efficacy and predictability; for prophylactic CXL with the LASIK procedure, the higher myopia, the more VA stability after CXL.

### Corneal stability - functional parameters

Based on our meta-analysis, CXL induced a significant stabilizing effect on VA within 12 months following laser refractive surgery technique, that is, UDVA and CDVA stability induced by prophylactic CXL persisted for at least 12 months post-refractive surgery. According to a large retrospective comparative study by Randleman et al., [5, 37] approximately 50% of ectasia cases occur within the first year after refractive surgery technique, so stabilizing the cornea for the first 12 months post-operatively may be critical in clinical practice to reduce the subsequent ectasia risk. In addition, because ectasia is a progressive disorder and CXL could only partially reverse its progression [8], prophylactic CXL before vision deterioration may be preferable given that the current evidence is limited for the effect of CXL longer than 12 months. Besides, the soaking of riboflavin in prophylactic CXL plus the LASIK procedure was performed directly on the exposed stroma after the flap was created, indicating that riboflavin diffusion may not be reduced by the flap in the case of post-LASIK corneal ectasia [38]. Thus, the more prolonged stabilizing effect of prophylactic CXL compared to CXL for post-LASIK keratectasia is promising and future longer-term RCTs are warranted to validate the maintenance effect of prophylactic CXL with laser refractive surgery technique.

### Corneal stability – factors influencing treatment effects

Furthermore, high myopia and undergoing the LASIK procedure as the refractive were the two main factors associated with more visual stability after prophylactic CXL.

### High myopia

A prior meta-analysis [38] demonstrated that highly myopic eyes have significantly lower corneal hysteresis (CH) and corneal resistant factor (CRF) values, possibly due to differences in scleral structures, including tissue loss, altered distribution of collagen fibers, and collagen degradation contributing to differing corneal biomechanics.

### LASIK procedure

A systemic review by Guo et al. [39] including twenty-two studies reported that FS-LASIK or LASIK was inferior to in preserving corneal biomechanical strength after refractive surgery techniques based on more decreased CH and CRF. This is reasonable because the flap procedure is more likely to lower corneal biomechanics. However, LASIK procedures still have an indispensable role in those patients with astigmatism [40–42] or who do not have appropriate eye fixation (i.e., Bell’s phenomenon, large pre-operative angle kappa). 

In summary, previous articles indicate that high myopia and the LASIK procedure are associated with more corneal biomechanical weakening and our meta-analysis implies that eyes with a higher degree of corneal instability have more benefits of visual stabilizing through prophylactic CXL. In concordance with our findings, previous studies have shown CXL offers a more significant CH increase in the more biomechanically weakened cornea [43–46]. Wu and colleagues [47] suggested that highly myopic eyes have on average a thinner corneal flap indicating that the more anterior part of the stromal lamellae are affected, resulting in more weakened biomechanical strength after the LASIK procedure. Therefore, prophylactic CXL may be considered in patients who have highly myopic eyes or who are only suitable for the LASIK procedure to optimize the visual stabilizing effect after the intervention.

### Corneal stability – biomechanical parameters

Different from the results of VA above, the stability of the keratometry in the combined group was not superior to that in the laser refractive surgery technique alone group within 12 months post-operatively. However, to date, the correlation between the clinical and topographic changes of the cornea and improvements in VA has not been reported [48, 49]. Given that iatrogenic ectasia may occur over a long period of 10 years after refractive surgery technique, the 12-month and 24-month follow-up in most studies may not be long enough to reflect the stabilizing effect of keratometry in prophylactic CXL.

Besides, the present meta-analysis revealed that laser refractive surgery technique with prophylactic CXL achieved comparable stability in corneal thickness and ECD with laser refractive surgery technique alone in the follow-up of 12 months. It is well established that endothelial cell damage induced by UVA-irradiation is a potential complication in a thin cornea [50–52]*.* Therefore, considering this concept, no significant ECD decline after prophylactic CXL in our study suggests a relatively healthy cornea, compared to iatrogenic keratectasia, is adequate to attenuate the UVA-irradiation before it reaches the endothelium.

### Cost-effectiveness

Apart from CXL, no current treatments can mitigate the progression of keratectasia and that corneal transplantation (DALK and PKP) are the last resort in the case of severe vision loss. PKP is associated with poor quality of life and higher medical costs, thus legal issues may arise accompanied by patient dissatisfaction once the iatrogenic keratectasia occurred. Therefore, preventing iatrogenic keratectasia remains the primary goal but owing to the high cost of CXL, it is important to determine whether the individual and societal benefits of prophylactic CXL outweigh the costs. However, the cost-effectiveness of the CXL prophylaxis to prevent ectasia is limited by current evidence and remains questionable. Consequently, patient selection criteria for prophylactic cross-linking are critical and should be established before the intervention.

### Clinical implications

In current clinical practice, patients with moderate to high risk of ectasia are often contraindicated from receiving LASIK procedures. The alternative like phakic intraocular lens, covering more extended-range of myopia level, was considered a preferred choice over LASIK procedures for patients with moderate-to-severe myopia. However, higher surgery expertise and stricter candidate requirement (adequate anterior chamber depth (> 3.0 mm) and low astigmatism (< 2.5D)) contributed to the limited availability of phakic intraocular lens currently. Also, potential risks of glaucoma, cataract formation, and endothelial cell loss were another major concern and required longer-term follow-up than laser refractive surgery procedures. Therefore, prophylactic CXL could provide clinicians another choice by adding a relatively simple procedure to the laser refractive surgery procedures, especially for moderate-to-severe high myopic patients. Here, our meta-analysis demonstrated that patients with higher myopia, which partially represents a higher risk of ectasia, may benefit more from CXL concurrent with LASIK procedures. Despite the lack of clinical trials, our meta-analysis provides promising results so future high-quality RCTs are encouraged for further investigation of prophylactic CXL.

Our meta-analysis demonstrated a 0.04 logMAR less UDVA loss in prophylactic CXL with laser refractive surgery technique group. Lower UVA radiation power and shorter treatment duration in current prophylactic CXL protocols, compared to CXL protocols for keratoconus or iatrogenic keratectasia, may partially contribute to the small VA stability effect. Given analysis of more visual function (other than VA) is limited by current included studies (such as visual function score or questionnaire), future trials investigating more detailed visual function outcomes to evaluate the efficacy and safety are warranted.

### Strengths and limitations

This is the first systemic review and meta-analysis focusing on the corneal stability of prophylactic CXL with laser refractive surgery technique for post-refractive ectasia prevention. In this meta-analysis, we analyzed postoperative changes of several surrogate parameters to represent corneal stability and specifically analyze these effects at different time points. However, there were still some limitations. First, apart from the small number of relevant RCTs, especially those performing PRK and KLEx as refractive surgery techniques, different study designs and populations with varied corneal CXL protocols such as irradiation intensity, exposure duration, and riboflavin concentrations may all potentially lead to substantial clinical heterogeneity. Second, most studies did not report detailed biomechanical and topography parameters [surface regularity index (SRI), surface asymmetry index (SAI), and keratoconus prediction index (KPI)], which provide direct evidence for early detection of post-refractive ectasia. Finally, the relatively short follow-up periods in the current studies (only one had provided 24 months of follow-up data) mean that longer-term studies assessing the effect on corneal stability after prophylactic CXL are required to validate the persistent benefits of prophylactic CXL with laser refractive surgery technique. However, even with these limitations, securing initial stability of the cornea is still an indispensable prerequisite to avoid future iatrogenic keratectasia from occurring. So, our finding of better initial 12-month VA stability after prophylactic CXL with laser refractive surgery technique in high myopic eyes may shed a light on the clinical practice and provide more treatment options for the ophthalmologists in the future.

## Conclusion

In conclusion, prophylactic CXL with laser refractive surgery technique offers significantly better visual stability in the 12-month post-operative period in myopic eyes compared to laser refractive surgery technique alone. This beneficial effect seems to be more substantial in highly myopic patients undergoing LASIK procedures. From a safety perspective, this meta-analysis indicates that prevention is better than treatment for post-refractive keratectasia.

## Supplementary Information

Below is the link to the electronic supplementary material.

Supplementary file1 (DOCX 15 KB)

Supplementary file2 (DOCX 17 KB)

Supplementary file3 (DOCX 21 KB)

Supplementary file4 (DOCX 40 KB)

Supplementary file5 (DOCX 322 KB)

Supplementary file6 (DOCX 1477 KB)

Supplementary file7 (DOCX 3744 KB)

Supplementary file8 (DOCX 4317 KB)

Supplementary file9 (DOCX 2462 KB)

Supplementary file10 (DOCX 3279 KB)

Supplementary file11 (DOCX 4132 KB)

Supplementary file12 (DOCX 2904 KB)

Supplementary file13 (DOCX 16184 KB)

Supplementary file14 (DOCX 13 KB)

## Abbreviations

CXL: Corneal cross-linking
LASIK: Laser-assisted in situ keratomileusis
KLEx: Keratorefractive Lenticule Extraction
SMILE: Small Incision Lenticule Extraction
PRK: Photorefractive Keratectomy
UDVA: Uncorrected distance visual acuity
CDVA: Corrected distance visual acuity
