# Supplementary material for: Corneal stability comparison between prophylactic cross-linking with laser refractive surgery technique versus laser refractive surgery technique alone for myopia: a meta-analysis
Source: Graefes Arch Clin Exp Ophthalmol. 2025 Sep 11;263(11):3037–52. doi: 10.1007/s00417-025-06833-6 (PMC12675695; doi:10.1007/s00417-025-06833-6)
Supplement: Supplementary file 1 — Supplementary file1 (DOCX 15 KB) [file 417_2025_6833_MOESM1_ESM.docx]

**Online resource 1. Mesh Term**

**e. Methods1. Mesh term**

| Database | Search Strategy | Results |
| --- | --- | --- |
| PUBMED | ((("keratomileusis, Laser in situ"[Mesh] OR “LASIK”[tiab]) OR ("small incision lenticule extraction” [tiab] OR “SMILE”[tiab]) OR ("Refractive Surgical Procedures"[Mesh] OR "Refractive"[tiab]) AND ("cross-linking"[tiab] OR “crosslinking”[tiab] OR “CXL”[tiab] OR “riboflavin”[tiab] OR “Xtra”[tiab])) AND ("Myopia, Degenerative "[mesh] OR "Myopia"[mesh] OR "Myopia"[tiab] OR "Myopic"[tiab]) | 167 references |
| EMBASE | ('laser-assisted in-situ keratomileusis'/exp OR 'laser-assisted in-situ keratomileusis' OR 'lasik'/exp OR 'lasik' OR 'laser-assisted in-situ keratomileusis':ab,ti OR 'laser in-situ keratomileusis':ab,ti OR 'lasik':ab,ti OR 'small incision lenticule extraction'/exp OR 'small incision lenticule extraction' OR 'smile'/exp OR 'smile' OR 'small incision lenticule extraction':ab,ti OR 'smile':ab,ti) AND ('cross-linking'/exp OR 'cross-linking' OR 'crosslinking'/exp OR 'crosslinking' OR 'cxl' OR 'xtra' OR 'riboflavin'/exp OR 'riboflavin' OR 'cross-linking':ab,ti OR 'crosslinking':ab,ti OR 'cxl':ab,ti OR 'xtra':ab,ti OR 'riboflavin':ab,ti) AND ('myopia'/exp OR 'myopia' OR 'myopic' OR 'myopia':ab,ti OR 'myopic':ab,ti) | 103 references |
| WEB OF SCIENCE | **#1 (“laser-assisted in-situ keratomileusis” OR “laser intrastromal keratomileusis” OR “LASIK” OR “small incision lenticule extraction” OR “SMILE” OR “refractive”)** (Topic)or (**“laser-assisted in-situ keratomileusis” OR “laser intrastromal keratomileusis” OR “LASIK” OR “small incision lenticule extraction” OR “SMILE” OR “refractive”)** (Title)  **#2 (“crosslinking” OR “cross-linking” OR “riboflavin” OR “CXL” OR “Xtra”)** (Topic) or **(“crosslinking” OR “cross-linking” OR “riboflavin” OR “CXL” OR “Xtra”)** (Title)  **#3 (“myopia” OR “myopic”)** (Topic) or **(“myopia” OR “myopic”)** (Title)  #1 AND #2 AND #3 | 214 references |
| COCHRANE | (“laser-assisted in-situ keratomileusis” or “laser intrastromal keratomileusis” or “LASIK” or “small incision lenticule extraction” or “SMILE” or “refractive”:ti,ab) AND ("cross-linking" or "crosslinking" or "CXL" or "riboflavin" or "Xtra":ti,ab) AND ("Myopia" or “Myopic”: ti,ab)  MeSH descriptor: [Myopia] this term only  MeSH descriptor: [Keratomileusis, Laser In Situ] explode all trees | 31 trials |
| ClinicalTrials.gov | (cross-linking OR CXL) AND Myopia | 8 trials |
