# Supplementary material for: Corneal stability comparison between prophylactic cross-linking with laser refractive surgery technique versus laser refractive surgery technique alone for myopia: a meta-analysis
Source: Graefes Arch Clin Exp Ophthalmol. 2025 Sep 11;263(11):3037–52. doi: 10.1007/s00417-025-06833-6 (PMC12675695; doi:10.1007/s00417-025-06833-6)
Supplement: Supplementary file 2 — Supplementary file2 (DOCX 17 KB) [file 417_2025_6833_MOESM2_ESM.docx]

| Initial Data | Conversion Formula ^1,2^ | Final Data |
| --- | --- | --- |
| SD of the mean in *X* at two follow-up periods (t1, t2) | ${SD}_{E/C,change}=\sqrt{{{SD}_{E/C, t1}}^{2}+{{SD}_{E/C, t2}}^{2}-(2*Corr*{SD}_{E/C,t1}*{SD}_{E/C,t2})} ADDIN ZOTERO\_TEMP$  Where ${SD}_{\frac{E}{C},change}$= standard deviation of the mean change of *X*  ${SD}_{\frac{E}{C},t1}$= standard deviation of the mean *X* at follow-up period(t1)  ${SD}_{\frac{E}{C},t2}$= standard deviation of the mean *X* at follow-up period(t2)  $Corr$ = correlation coefficient | SD of the mean change in *X* at two follow-up periods (t1, t2) |
| SE of the mean change in *X* | $SE=\frac{\sigma_{X change}}{\sqrt{n}}$  Where $\sigma_{X change}$ = standard deviation of mean *X* change  n = sample size | SD of the mean change in *X* |
| Group1 and Group2 means and SDs | $M_{combined}=\frac{N_{1}M_{1}+N_{2}M_{2}}{N_{1}+N_{2}}$  ${SD}_{combined}=\sqrt{\frac{(N_{1}-1){{SD}_{1}}^{2}+(N_{2}-1){{SD}_{2}}^{2}+\frac{N_{1}N_{2}}{N_{1}+N_{2}}({M_{1}}^{2}+{M_{2}}^{2}-2M_{1}M_{2})}{N_{1}+N_{2}-1}}$  Where $N_{1}=Group 1 same size ; N_{2}=Group 2 same size$  $M_{1}=Group 1 mean ; N_{2}=Group 2 mean$  $\mathrm{SD}_{1}=Group 1 standard deviation ; \mathrm{SD}_{2}=Group 2 standard deviation$ | Combined mean and SD. |
| Note: *X* = Continuous outcomes of our outcome of interest: UDVA, CDVA, K, MRSE, CT, ECD  Abbreviations: SD, standard deviation; SE, standard error | | |

**Online resource 2. Data Conversion Formula**

**eMethod2. Data Conversion Formulas**
