# Supplementary material for: Corneal stability comparison between prophylactic cross-linking with laser refractive surgery technique versus laser refractive surgery technique alone for myopia: a meta-analysis
Source: Graefes Arch Clin Exp Ophthalmol. 2025 Sep 11;263(11):3037–52. doi: 10.1007/s00417-025-06833-6 (PMC12675695; doi:10.1007/s00417-025-06833-6)
Supplement: Supplementary file 3 — Supplementary file3 (DOCX 21 KB) [file 417_2025_6833_MOESM3_ESM.docx]

**Online resource 3. Risk of Bias in Randomized Trials, ROB2 Tool**

**eTable 3. Risk of Bias in randomized trials, ROB 2 tool** ^1,2^

| Risk of bias due to | | | | | | |
| --- | --- | --- | --- | --- | --- | --- |
| Outcome/ Studies | Randomization process | Deviations from the intended interventions ^b^ | Missing outcome data | Measurement of the outcome ^e^ | Selection of the reported result ^e^ | Overall bias |
| **Stability – UDVA change (logMAR)** | | | | | | |
| Kohnen et al (2020) | Low risk | Low risk | Low risk | Low risk ^d^ | Low risk | Low risk |
| Dong et al (2022) | Low risk | Low risk | Low risk | Some concern^c^ | Low risk | Low risk |
| **Stability – CDVA change (logMAR)** | | | | | | |
| Kohnen et al (2020) | Low risk | Low risk | Low risk | Low risk ^d^ | Low risk | Low risk |
| Dong et al (2022) | Low risk | Low risk | Low risk | Some concern^c^ | Low risk | Low risk |
| **Stability – MRSE change (D)** | | | | | | |
| Kohnen et al (2020) | Low risk | Low risk | Low risk | Low risk ^d^ | Low risk | Low risk |
| Dong et al (2022) | Low risk | Low risk | Low risk | Low risk | Low risk | Low risk |
| **Stability – Keratometry change** | | | | | | |
| Dong et al (2022) | Low risk | Low risk | Low risk | Low risk | Low risk | Low risk |
| **Stability – corneal thickness change (um)** | | | | | | |
| Kohnen et al (2020) | Low risk | Low risk | Low risk | Low risk ^d^ | Low risk | Low risk |
| **Stability – ECD change (cells/mm^2^)** | | | | | | |
| Kohnen et al (2020) | Low risk | Low risk | Low risk | Low risk ^d^ | Low risk | Low risk |
| Dong et al (2022) | Low risk | Low risk | Low risk | Low risk | Low risk | Low risk |
| **Efficacy –% of eyes achieving UDVA of 20/20 or better / 20/25 or better** | | | | | | |
| Kohnen et al (2020) | Low risk | Low risk | Low risk | Low risk ^d^ | Low risk | Low risk |
| Kanellopoulos et al (2015) | Some concern^a^ | Low risk | Low risk | Some concern^c^ | Low risk | Some concern |
| Dong et al (2022) | Low risk | Low risk | Low risk | Some concern^c^ | Low risk | Low risk |
| **Predictability – % of eyes within 1.0 D or 0.5 D of attempted refractive correction** | | | | | | |
| Kohnen et al (2020) | Low risk | Low risk | Low risk | Low risk ^d^ | Low risk | Low risk |
| Kanellopoulos et al (2015) | Some concern^a^ | Low risk | Low risk | Low risk | Low risk | Some concern |
| Dong et al (2022) | Low risk | Low risk | Low risk | Low risk | Low risk | Low risk |
| **Safety – % of eyes with one or more lines of loss in CDVA** | | | | | | |
| Kohnen et al (2020) | Low risk | Low risk | Low risk | Low risk ^d^ | Low risk | Low risk |
| Kanellopoulos et al (2015) | Some concern^a^ | Low risk | Low risk | Some concern^c^ | Low risk | Some concern |
| Dong et al (2022) | Low risk | Low risk | Low risk | Some concern^c^ | Low risk | Low risk |

Abbreviation; UDVA, uncorrected distance visual acuity; CDVA, corrected distance visual acuity; MRSE, manifest refraction spherical equivalent; ECD, endothelial cell density.

a. no information about whether the allocation sequence is concealed

b. surgical intervention (CXL + refractive surgery) administered once, so that imperfect adherence is not possible, and all or most participants received the assigned intervention.

c. outcome assessors aware of the intervention; assessment of UDVA and CDVA are participant-reported outcomes but was not likely to be influenced by accessors judgement.

d. outcome assessors were blinded to the intervention

e. Kanellopoulos et al measured all outcomes (VA, MRSE, corneal thickness, ECD) at multiple time points (post-operative 1,3,6, 12-month), but only reported outcomes at the specific time point (post-operative 12-month).
