# Supplementary material for: Corneal stability comparison between prophylactic cross-linking with laser refractive surgery technique versus laser refractive surgery technique alone for myopia: a meta-analysis
Source: Graefes Arch Clin Exp Ophthalmol. 2025 Sep 11;263(11):3037–52. doi: 10.1007/s00417-025-06833-6 (PMC12675695; doi:10.1007/s00417-025-06833-6)
Supplement: Supplementary file 4 — Supplementary file4 (DOCX 40 KB) [file 417_2025_6833_MOESM4_ESM.docx]

**Online resource 4. Risk Of Bias for Observational Studies, Newcastle-Ottawa Scale**

**eTable 4-1. Quality assessment using the Newcastle-Ottawa Scale for observational studies – UDVA Change**

| **Study** | **Selection** | | | | **Comparability ^a^** | **Outcome** | | |  |
| --- | --- | --- | --- | --- | --- | --- | --- | --- | --- |
|  | Representative of the exposed cohort | Selection of the unexposed cohort | Ascertainment of exposure | Demonstration that outcome of interest was not present at start of the study | Comparability of cohorts on the basis of the design or analysis | Assessment of outcome | Was follow-up long enough for outcomes to occur | Adequacy of follow-up for cohorts | Scores |
| Zhang et al (2022) | ☆ | ☆ | ☆ | ☆ | - | ☆ | ☆ | -^c^ | 6 |
| Brar et al (2022) | ☆ | ☆ | ☆ | ☆ | - ^b^ | ☆ | ☆ | -^c^ | 6 |
| Osman et al (2019) | ☆ | ☆ | ☆ | ☆ | - ^b^ | ☆ | ☆ | ☆^d^ | 7 |
| Lee et al (2017) | ☆ | ☆ | ☆ | ☆ | - ^b^ | ☆ | ☆ | ☆^d^ | 7 |
| Tomita **e**t al (2014) | ☆ | ☆ | ☆ | ☆ | - | ☆ | ☆ | ☆^d^ | 7 |

a. A maximum of two stars can be given for Comparability: one for age and sex, the other for other controlled factors.

b. Trials only have statements of no baseline patient/ocular characteristics difference between groups.

c. No description of the follow-up rate.

d. Complete follow up.

**eTable 4-2. Quality assessment using the Newcastle-Ottawa Scale for observational studies – CDVA change**

| **Study** | **Selection** | | | | **Comparability ^a^** | **Outcome** | | |  |
| --- | --- | --- | --- | --- | --- | --- | --- | --- | --- |
|  | Representative of the exposed cohort | Selection of the unexposed cohort | Ascertainment of exposure | Demonstration that outcome of interest was not present at start of the study | Comparability of cohorts on the basis of the design or analysis | Assessment of outcome | Was follow-up long enough for outcomes to occur | Adequacy of follow-up for cohorts | Scores |
| Zhang et al (2022) |  |  |  |  |  |  |  |  |  |
| Osman et al (2019) | ☆ | ☆ | ☆ | ☆ | - ^b^ | ☆ | ☆ | ☆^c^ | 7 |
| Tomita **e**t al (2014) | ☆ | ☆ | ☆ | ☆ | - | ☆ | ☆ | ☆ | 7 |

a. A maximum of two stars can be given for Comparability: one for age and sex, the other for other controlled factors.

b. Trials only have statements of no baseline patient/ocular characteristics difference between groups.

c. Complete follow up.

**eTable 4-3. Quality assessment using the Newcastle-Ottawa Scale for observational studies – Keratometry change**

| **Study** | **Selection** | | | | **Comparability ^a^** | **Outcome** | | |  |
| --- | --- | --- | --- | --- | --- | --- | --- | --- | --- |
|  | Representative of the exposed cohort | Selection of the unexposed cohort | Ascertainment of exposure | Demonstration that outcome of interest was not present at start of the study | Comparability of cohorts on the basis of the design or analysis | Assessment of outcome | Was follow-up long enough for outcomes to occur | Adequacy of follow-up for cohorts | Scores |
| Brar et al (2022) | ☆ | ☆ | ☆ | ☆ | - ^b^ | ☆ | ☆ | -^c^ | 6 |
| Chabib et al (2022) | ☆ | ☆ | ☆ | ☆ | - ^b^ | ☆ | ☆ | ☆^d^ | 7 |
| Osman et al (2019) |  |  |  |  |  |  |  |  |  |
| Tomita **e**t al (2014) | ☆ | ☆ | ☆ | ☆ | - | ☆ | ☆ | ☆ | 7 |

a. A maximum of two stars can be given for Comparability: one for age and sex, the other for other controlled factors.

b. Trials only have statements of no baseline patient/ocular characteristics difference between groups.

c. Follow up rate less than 80% and no description of those lost

d. Complete follow up.

**eTable 4-4. Quality assessment using the Newcastle-Ottawa Scale for observational studies – MRSE change**

| **Study** | **Selection** | | | | **Comparability ^a^** | **Outcome** | | |  |
| --- | --- | --- | --- | --- | --- | --- | --- | --- | --- |
|  | Representative of the exposed cohort | Selection of the unexposed cohort | Ascertainment of exposure | Demonstration that outcome of interest was not present at start of the study | Comparability of cohorts on the basis of the design or analysis | Assessment of outcome | Was follow-up long enough for outcomes to occur | Adequacy of follow-up for cohorts | Scores |
| Brar et al (2022) | ☆ | ☆ | ☆ | ☆ | - ^b^ | ☆ | ☆ | -^c^ | 6 |
| Chabib et al (2022) | ☆ | ☆ | ☆ | ☆ | - ^b^ | ☆ | ☆ | ☆^d^ | 7 |
| Zhang et al (2022) | ☆ | ☆ | ☆ | ☆ | - | ☆ | ☆ | -^c^ | 6 |
| Liu et al (2021) | ☆ | ☆ | ☆ | ☆ | - | ☆ | ☆ | ☆^d^ | 7 |
| Osman et al (2019) | ☆ | ☆ | ☆ | ☆ | - ^b^ | ☆ | ☆ | ☆^d^ | 7 |
| Lee et al (2017) | ☆ | ☆ | ☆ | ☆ | - ^b^ | ☆ | ☆ | ☆^d^ | 7 |
| Tomita et al (2014) | ☆ | ☆ | ☆ | ☆ | - | ☆ | ☆ | ☆ | 7 |

a. A maximum of two stars can be given for Comparability: one for age and sex, the other for other controlled factors.

b. Trials only have statements of no baseline patient/ocular characteristics difference between groups.

c. No description of the follow-up rate.

d. Complete follow up.

**eTable 4-5. Quality assessment using the Newcastle-Ottawa Scale for observational studies – CT change**

| **Study** | **Selection** | | | | **Comparability ^a^** | **Outcome** | | |  |
| --- | --- | --- | --- | --- | --- | --- | --- | --- | --- |
|  | Representative of the exposed cohort | Selection of the unexposed cohort | Ascertainment of exposure | Demonstration that outcome of interest was not present at start of the study | Comparability of cohorts on the basis of the design or analysis | Assessment of outcome | Was follow-up long enough for outcomes to occur | Adequacy of follow-up for cohorts | Scores |
| Osman et al (2019) | ☆ | ☆ | ☆ | ☆ | - ^b^ | ☆ | ☆ | ☆^c^ | 7 |

a. A maximum of two stars can be given for Comparability: one for age and sex, the other for other controlled factors.

b. Trials only have statements of no baseline patient/ocular characteristics difference between groups.

c. Complete follow up.

**eTable 4-6. Quality assessment using the Newcastle-Ottawa Scale for observational studies – ECD change**

| **Study** | **Selection** | | | | **Comparability ^a^** | **Outcome** | | |  |
| --- | --- | --- | --- | --- | --- | --- | --- | --- | --- |
|  | Representative of the exposed cohort | Selection of the unexposed cohort | Ascertainment of exposure | Demonstration that outcome of interest was not present at start of the study | Comparability of cohorts on the basis of the design or analysis | Assessment of outcome | Was follow-up long enough for outcomes to occur | Adequacy of follow-up for cohorts | Scores |
| Osman et al (2019) | ☆ | ☆ | ☆ | ☆ | - ^b^ | ☆ | ☆ | ☆^d^ | 7 |
| Tomita **e**t al (2014) | ☆ | ☆ | ☆ | ☆ | - | ☆^c^ | ☆ | ☆ | 8 |

a. A maximum of two stars can be given for Comparability: one for age and sex, the other for other controlled factors.

b. Trials only have statements of no baseline patient/ocular characteristics difference between groups.

c. Independent blinding assessment: two physicians evaluated the corneal tissue morphology on the images without knowing which eye had CXL.

d. Complete follow up.

**eTable 4-7. Quality assessment using the Newcastle-Ottawa Scale for observational studies – Efficacy**

| **Study** | **Selection** | | | | **Comparability ^a^** | **Outcome** | | |  |
| --- | --- | --- | --- | --- | --- | --- | --- | --- | --- |
|  | Representative of the exposed cohort | Selection of the unexposed cohort | Ascertainment of exposure | Demonstration that outcome of interest was not present at start of the study | Comparability of cohorts on the basis of the design or analysis | Assessment of outcome | Was follow-up long enough for outcomes to occur | Adequacy of follow-up for cohorts | Scores |
| Brar et al (2022) | ☆ | ☆ | ☆ | ☆ | - ^b^ | ☆ | ☆ | -^c^ | 6 |
| Chabib et al (2022) | ☆ | ☆ | ☆ | ☆ | - ^b^ | ☆ | ☆ | ☆^d^ | 7 |
| Liu et al (2021) | ☆ | ☆ | ☆ | ☆ | - | ☆ | ☆ | ☆^d^ | 7 |
| Osman et al (2019) | ☆ | ☆ | ☆ | ☆ | - ^b^ | ☆ | ☆ | ☆^d^ | 7 |
| Sachdev et al (2018) | ☆ | ☆ | ☆ | ☆ | - | ☆ | ☆ | ☆^d^ | 7 |
| Lee et al (2017) | ☆ | ☆ | ☆ | ☆ | - ^b^ | ☆ | ☆ | ☆^d^ | 7 |
| Tomita **e**t al (2014) | ☆ | ☆ | ☆ | ☆ | - | ☆ | ☆ | ☆ | 8 |

a. A maximum of two stars can be given for Comparability: one for age and sex, the other for other controlled factors.

b. Trials only have statements of no baseline patient/ocular characteristics difference between groups.

c. No description of the follow-up rate.

d. Complete follow up.

**eTable 4-8. Quality assessment using the Newcastle-Ottawa Scale for observational studies – Predictability**

| **Study** | **Selection** | | | | **Comparability ^a^** | **Outcome** | | |  |
| --- | --- | --- | --- | --- | --- | --- | --- | --- | --- |
|  | Representative of the exposed cohort | Selection of the unexposed cohort | Ascertainment of exposure | Demonstration that outcome of interest was not present at start of the study | Comparability of cohorts on the basis of the design or analysis | Assessment of outcome | Was follow-up long enough for outcomes to occur | Adequacy of follow-up for cohorts | Scores |
| Brar et al (2022) | ☆ | ☆ | ☆ | ☆ | - ^b^ | ☆ | ☆ | -^d^ | 6 |
| Liu et al (2021) | ☆ | ☆ | ☆ | ☆ | - | ☆ | ☆ | ☆^e^ | 7 |
| Osman et al (2019) | ☆ | ☆ | ☆ | ☆ | - ^b^ | ☆ | ☆ | ☆^e^ | 7 |
| Lee et al (2017) | ☆ | ☆ | ☆ | ☆ | - ^b^ | ☆ | ☆ | ☆^e^ | 7 |
| Seiler et al (2015) | ☆ | ☆ | ☆ | ☆ | ☆☆^c^ | ☆ | ☆ | ☆ | 9 |
| Tomita **e**t al (2014) | ☆ | ☆ | ☆ | ☆ | - | ☆ | ☆ | ☆ | 8 |

a. A maximum of two stars can be given for Comparability: one for age and sex, the other for other controlled factors.

b. Trials only have statements of no baseline patient/ocular characteristics difference between groups.

c. Patient age, sex, and attempted refractive change were matched between the study group and the control group.

d. No description of the follow-up rate.

**eTable 4-9. Quality assessment using the Newcastle-Ottawa Scale for observational studies – Safety**

| **Study** | **Selection** | | | | **Comparability ^a^** | **Outcome** | | |  |
| --- | --- | --- | --- | --- | --- | --- | --- | --- | --- |
|  | Representative of the exposed cohort | Selection of the unexposed cohort | Ascertainment of exposure | Demonstration that outcome of interest was not present at start of the study | Comparability of cohorts on the basis of the design or analysis | Assessment of outcome | Was follow-up long enough for outcomes to occur | Adequacy of follow-up for cohorts | Scores |
| Chabib et al (2022) | ☆ | ☆ | ☆ | ☆ | - ^b^ | ☆ | ☆ | ☆^d^ | 7 |
| Liu et al (2021) | ☆ | ☆ | ☆ | ☆ | - | ☆ | ☆ | ☆^d^ | 7 |
| Osman et al (2019) | ☆ | ☆ | ☆ | ☆ | - ^b^ | ☆ | ☆ | ☆^d^ | 7 |
| Sachdev et al (2018) | ☆ | ☆ | ☆ | ☆ | - | ☆ | ☆ | ☆^d^ | 7 |
| Lee et al (2017) | ☆ | ☆ | ☆ | ☆ | - ^b^ | ☆ | ☆ | ☆^d^ | 7 |
| Seiler et al (2015) | ☆ | ☆ | ☆ | ☆ | ☆☆^c^ | ☆ | ☆ | ☆ | 9 |

a. A maximum of two stars can be given for Comparability: one for age and sex, the other for other controlled factors.

b. Trials only have statements of no baseline patient/ocular characteristics difference between groups.

c. Patient age, sex, and attempted refractive change were matched between the study group and the control group.

d. Complete follow up.
