# Supplementary material for: Corneal stability comparison between prophylactic cross-linking with laser refractive surgery technique versus laser refractive surgery technique alone for myopia: a meta-analysis
Source: Graefes Arch Clin Exp Ophthalmol. 2025 Sep 11;263(11):3037–52. doi: 10.1007/s00417-025-06833-6 (PMC12675695; doi:10.1007/s00417-025-06833-6)
Supplement: Supplementary file 5 — Supplementary file5 (DOCX 322 KB) [file 417_2025_6833_MOESM5_ESM.docx]

**Online resource 5. Sensitivity Analysis – Outlier Removal**

We additionally performed a sensitivity analysis by removing the study by Dong et al. as the outlier given the confidential interval of the study doesn’t overlap with the overall confidential interval, which produced a more significantly less UDVA and CDVA loss in prophylactic CXL with laser refractive surgery technique than those receiving laser refractive surgery technique alone.

**eFigure 5.1 UDVA**

**
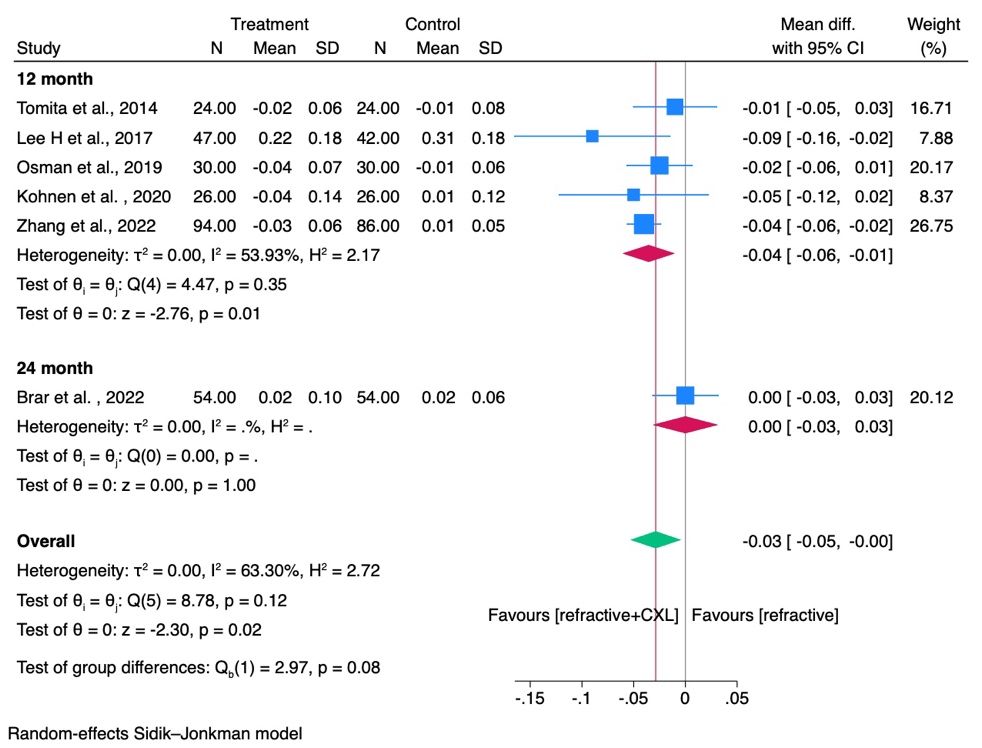
**

**eFigure 5.2 CDVA**

**
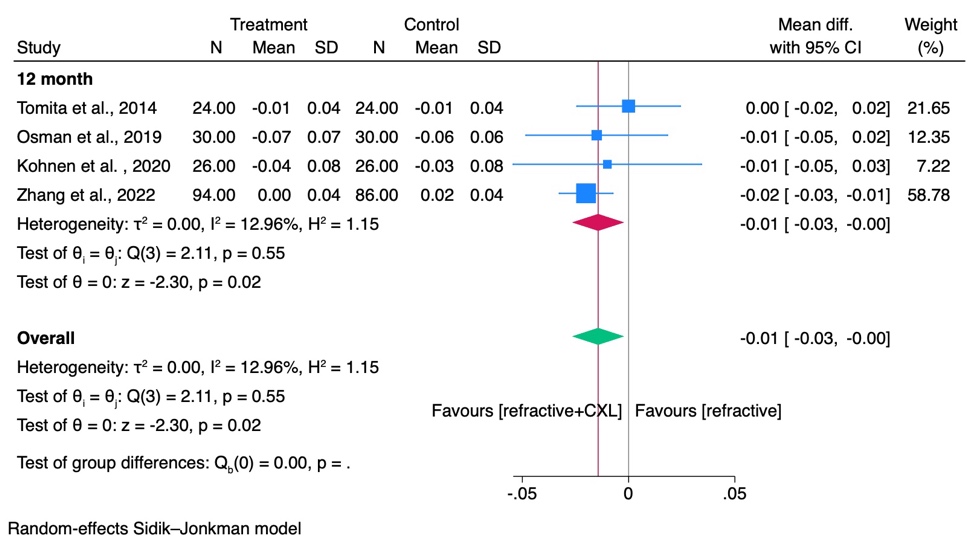
**
