# Supplementary material for: Corneal stability comparison between prophylactic cross-linking with laser refractive surgery technique versus laser refractive surgery technique alone for myopia: a meta-analysis
Source: Graefes Arch Clin Exp Ophthalmol. 2025 Sep 11;263(11):3037–52. doi: 10.1007/s00417-025-06833-6 (PMC12675695; doi:10.1007/s00417-025-06833-6)
Supplement: Supplementary file 6 — Supplementary file6 (DOCX 1477 KB) [file 417_2025_6833_MOESM6_ESM.docx]

**Online resource 6. Subgroup Analysis – Stratified by Study Design**


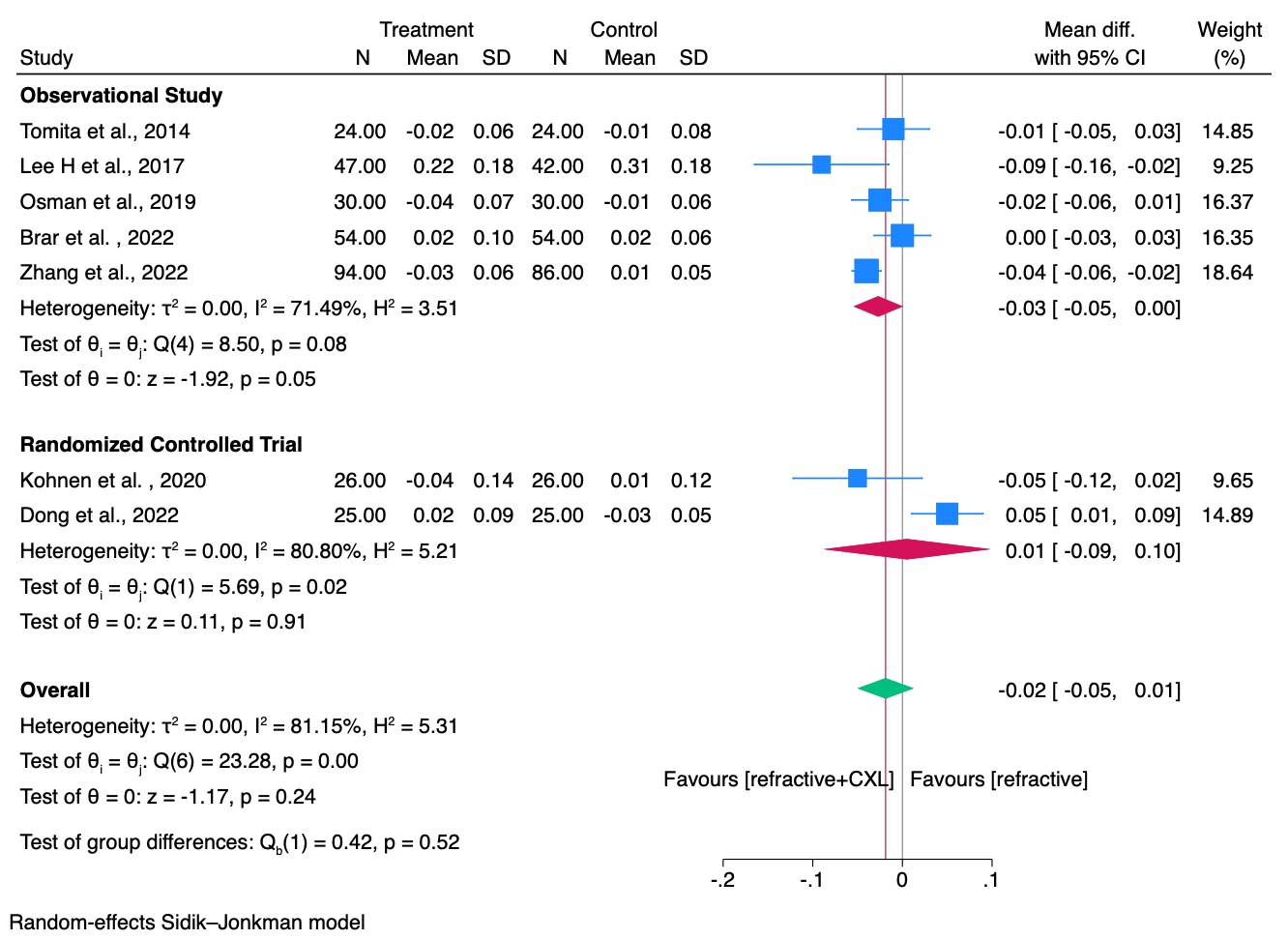


**eFigure 6.1 Forest plot for subgroup analysis comparing prophylactic CXL plus laser refractive surgery with laser refractive surgery alone on stability of UDVA in myopic patients by study design.** UDVA, uncorrected distance visual acuity; CXL, cross-linking; SD, standard deviations. Legend: The size of squares is proportional to the weight of each study. Horizontal lines indicate the 95% confidence intervals (CI) of mean difference estimate in each study; diamonds, the pooled estimate with 95% CI; N, the number of eyes at baseline; and SD, standard deviations.


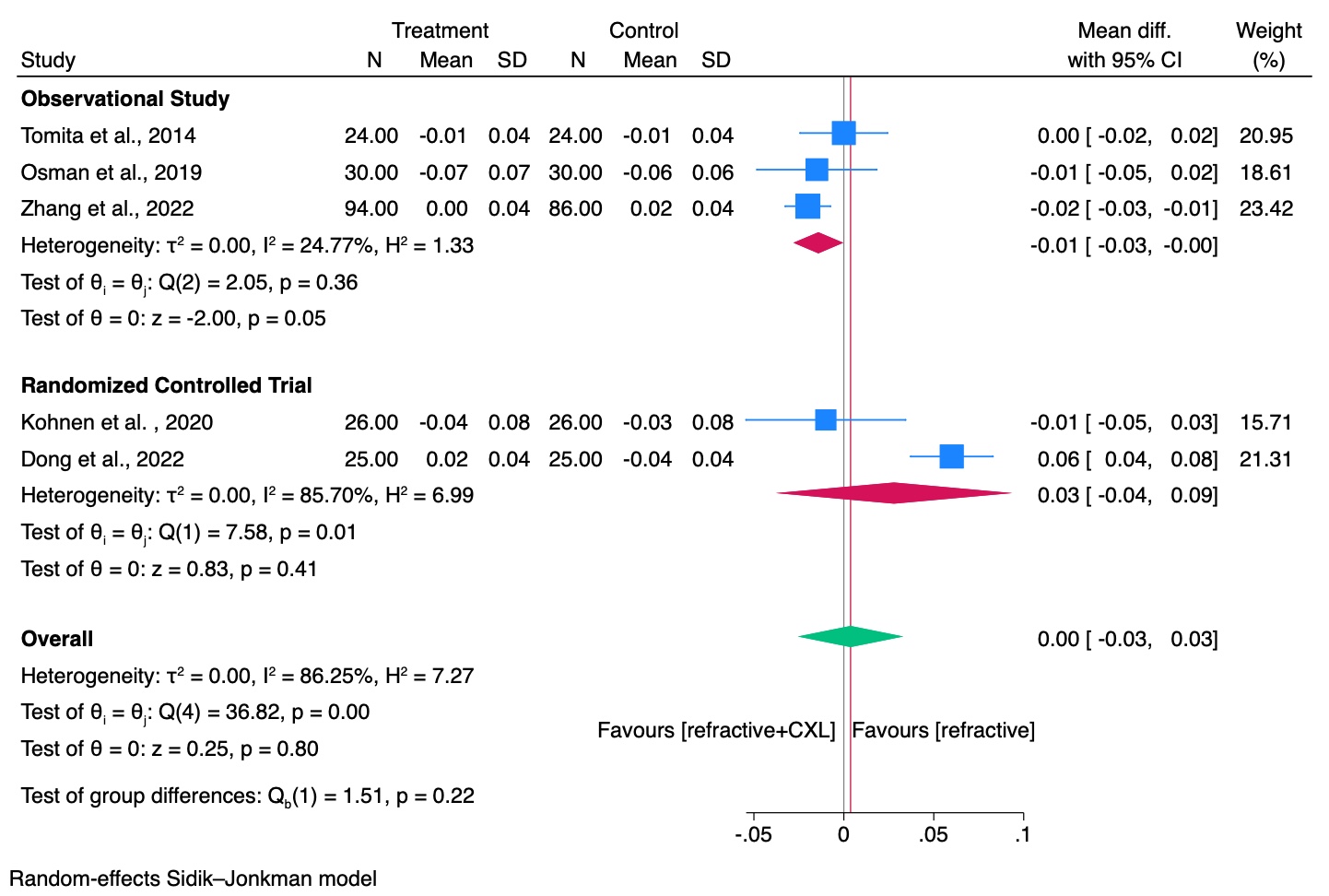


**eFigure 6.2 Forest plot for subgroup analysis comparing prophylactic CXL plus laser refractive surgery with laser refractive surgery alone on stability of CDVA in myopic patients by study design.** CDVA, corrected distance visual acuity; CXL, cross-linking; SD, standard deviations. Legend: The size of squares is proportional to the weight of each study. Horizontal lines indicate the 95% confidence intervals (CI) of mean difference estimate in each study; diamonds, the pooled estimate with 95% CI; N, the number of eyes at baseline; and SD, standard deviations.


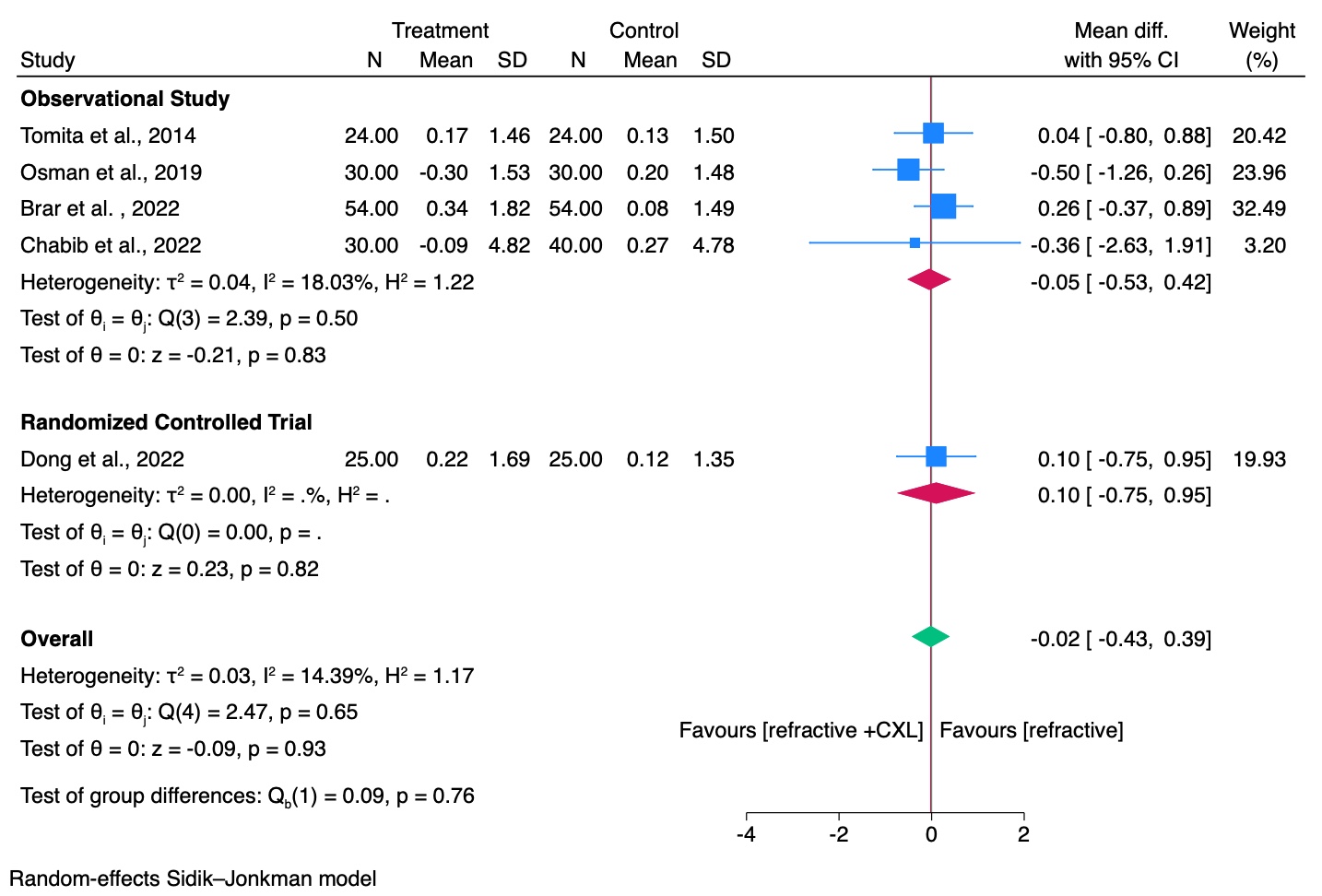


**eFigure 6.3 Forest plot for subgroup analysis comparing prophylactic CXL plus laser refractive surgery with laser refractive surgery alone on stability of keratometry in myopic patients by study design.** CXL, cross-linking; SD, standard deviations. Legend: The size of squares is proportional to the weight of each study. Horizontal lines indicate the 95% confidence intervals (CI) of mean difference estimate in each study; diamonds, the pooled estimate with 95% CI; N, the number of eyes at baseline; and SD, standard deviations.


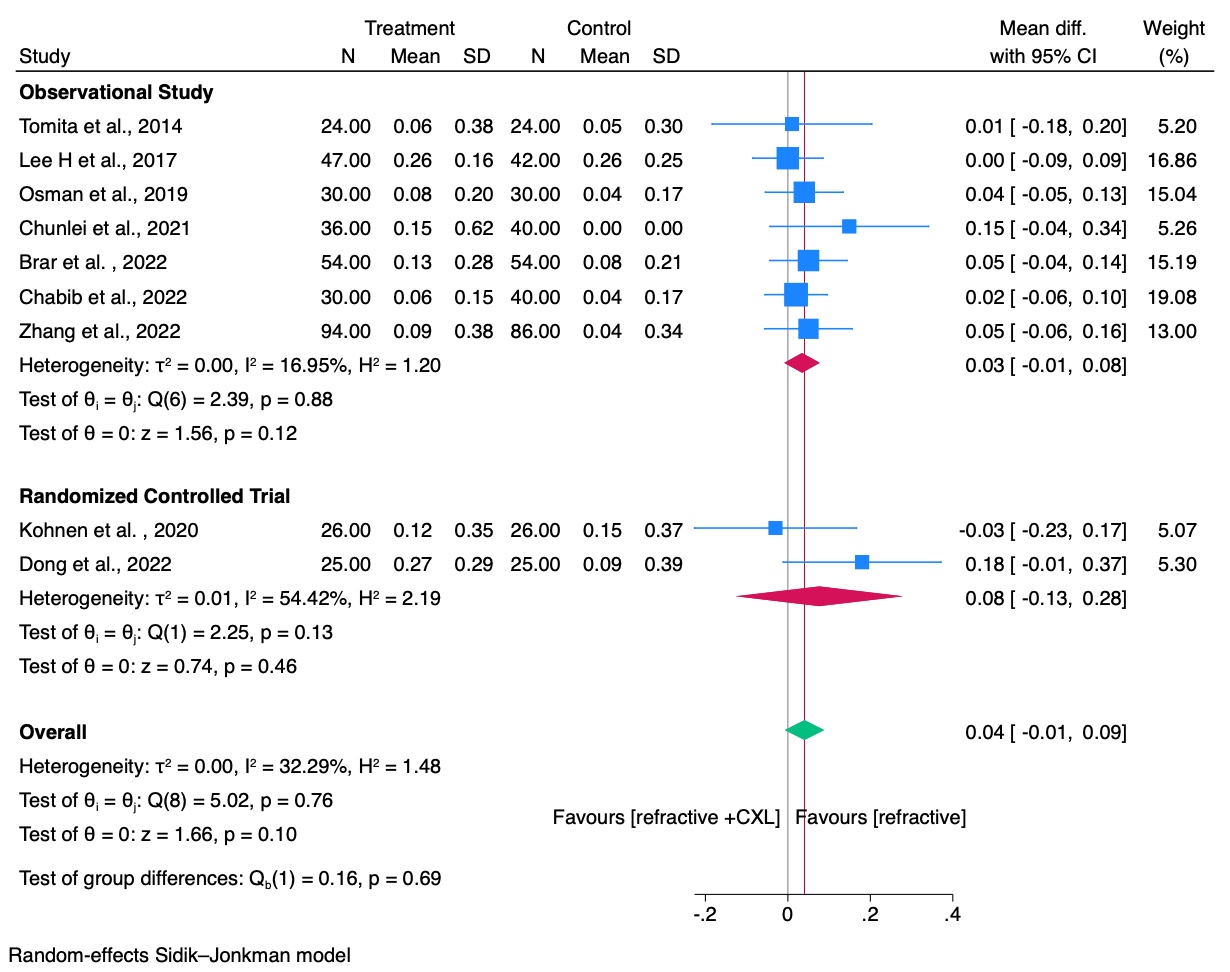


**eFigure 6.4 Forest plot for subgroup analysis comparing prophylactic CXL plus laser refractive surgery with laser refractive surgery alone on stability of MRSE in myopic patients by study design.** MRSE, manifest refractive spherical equivalent; CXL, cross-linking; SD, standard deviations. Legend: The size of squares is proportional to the weight of each study. Horizontal lines indicate the 95% confidence intervals (CI) of mean difference estimate in each study; diamonds, the pooled estimate with 95% CI; N, the number of eyes at baseline; and SD, standard deviations.


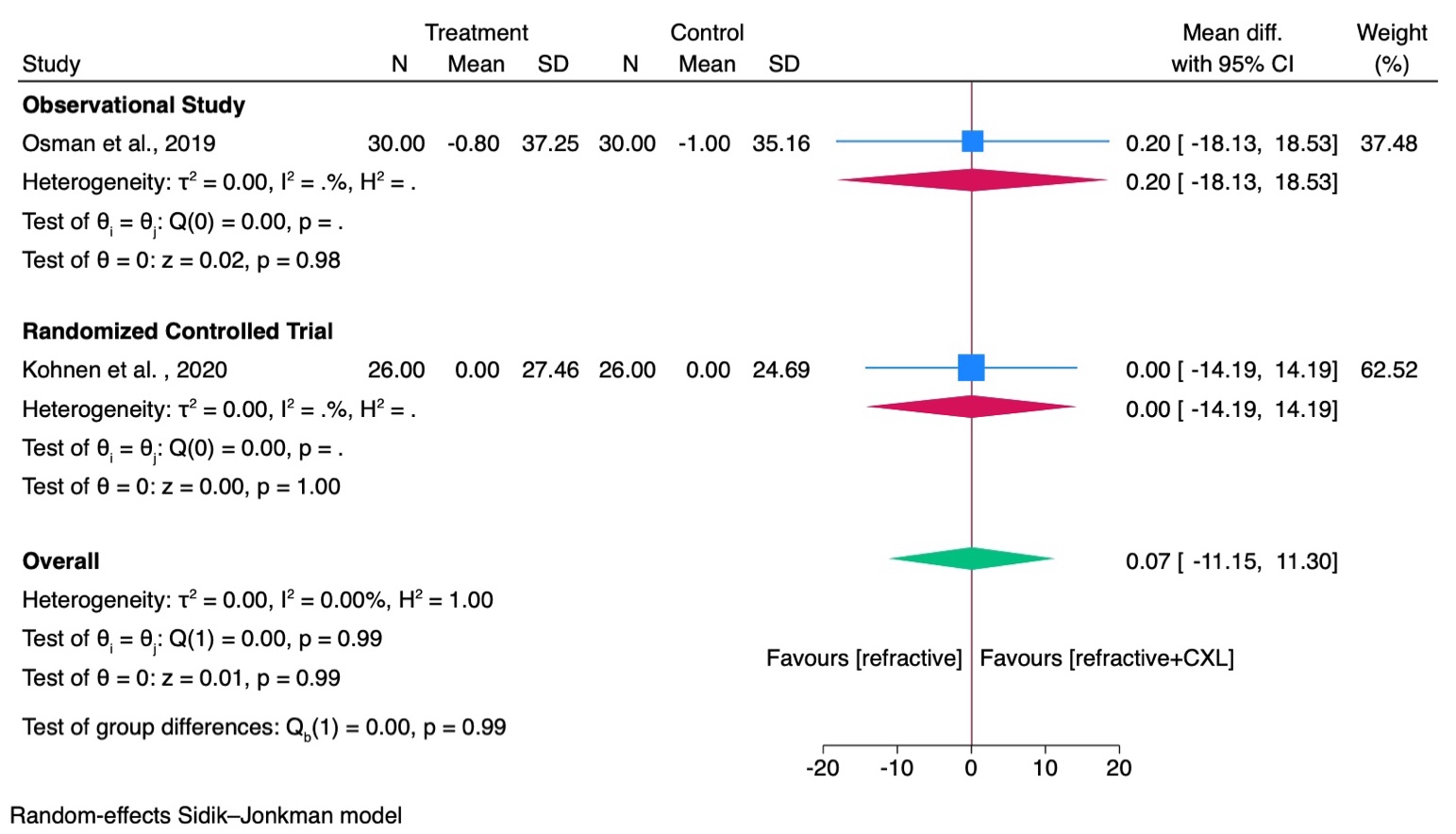


**eFigure 6.5 Forest plot for subgroup analysis comparing prophylactic CXL plus laser refractive surgery with laser refractive surgery alone on stability of corneal thickness in myopic patients by study design.** CXL, cross-linking; SD, standard deviations. Legend: The size of squares is proportional to the weight of each study. Horizontal lines indicate the 95% confidence intervals (CI) of mean difference estimate in each study; diamonds, the pooled estimate with 95% CI; N, the number of eyes at baseline; and SD, standard deviations.


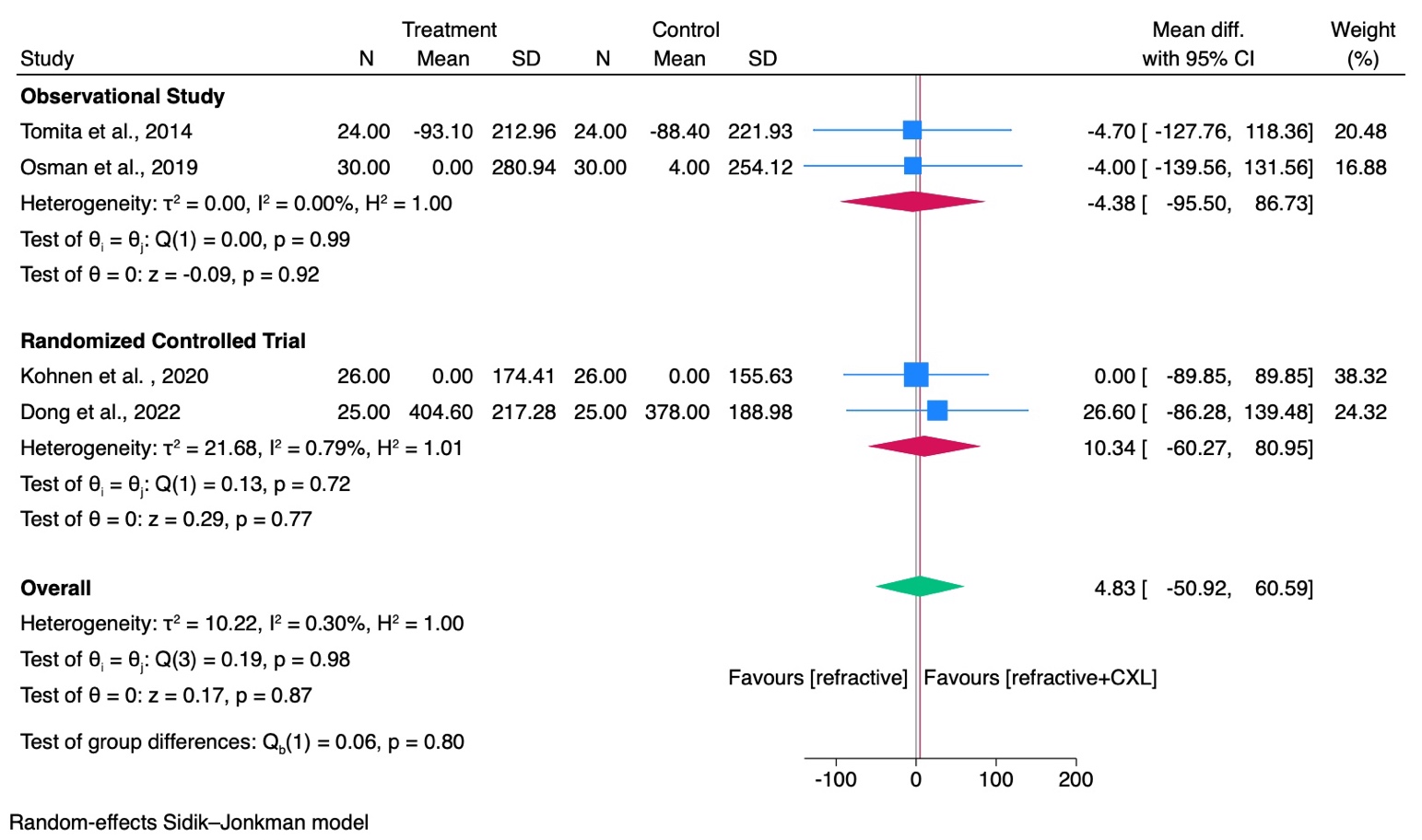


**eFigure 6.6 Forest plot for subgroup analysis comparing prophylactic CXL plus laser refractive surgery with laser refractive surgery alone on stability of ECD in myopic patients by study design.** ECD, endothelial cell count; CXL, cross-linking; SD, standard deviations. Legend: The size of squares is proportional to the weight of each study. Horizontal lines indicate the 95% confidence intervals (CI) of mean difference estimate in each study; diamonds, the pooled estimate with 95% CI; N, the number of eyes at baseline; and SD, standard deviations.
