# Supplementary material for: Corneal stability comparison between prophylactic cross-linking with laser refractive surgery technique versus laser refractive surgery technique alone for myopia: a meta-analysis
Source: Graefes Arch Clin Exp Ophthalmol. 2025 Sep 11;263(11):3037–52. doi: 10.1007/s00417-025-06833-6 (PMC12675695; doi:10.1007/s00417-025-06833-6)
Supplement: Supplementary file 8 — Supplementary file8 (DOCX 4317 KB) [file 417_2025_6833_MOESM8_ESM.docx]

**Online resource 8. Forest Plot of MRSE**

*
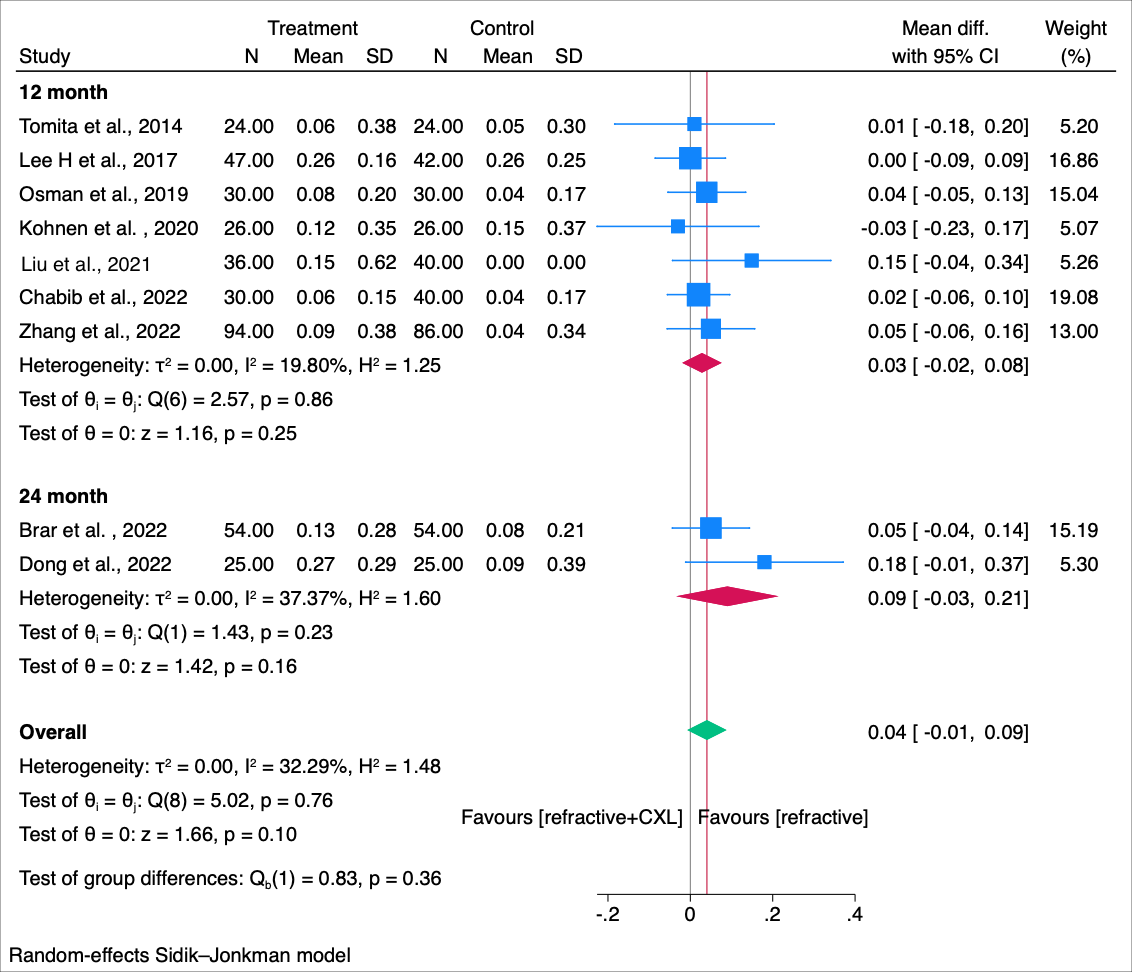
*

**eFigure 8. Forest plot for comparison of prophylactic CXL plus laser refractive surgery with laser refractive surgery alone on stability of MRSE in myopic patients at (A) 1-12 (B) 1-24 months.** CXL, cross-linking; MRSE, manifest refraction spherical equivalent; SD, standard deviations.

Legend: The size of squares is proportional to the weight of each study. Horizontal lines indicate the 95% confidence intervals (CI) of mean difference estimate in each study; diamonds, the pooled estimate with 95% CI; N, the number of eyes at baseline; and SD, standard deviation
