# Supplementary material for: Corneal stability comparison between prophylactic cross-linking with laser refractive surgery technique versus laser refractive surgery technique alone for myopia: a meta-analysis
Source: Graefes Arch Clin Exp Ophthalmol. 2025 Sep 11;263(11):3037–52. doi: 10.1007/s00417-025-06833-6 (PMC12675695; doi:10.1007/s00417-025-06833-6)
Supplement: Supplementary file 11 — Supplementary file11 (DOCX 4132 KB) [file 417_2025_6833_MOESM11_ESM.docx]

**Online resource 11. Forest Plot of Safety**


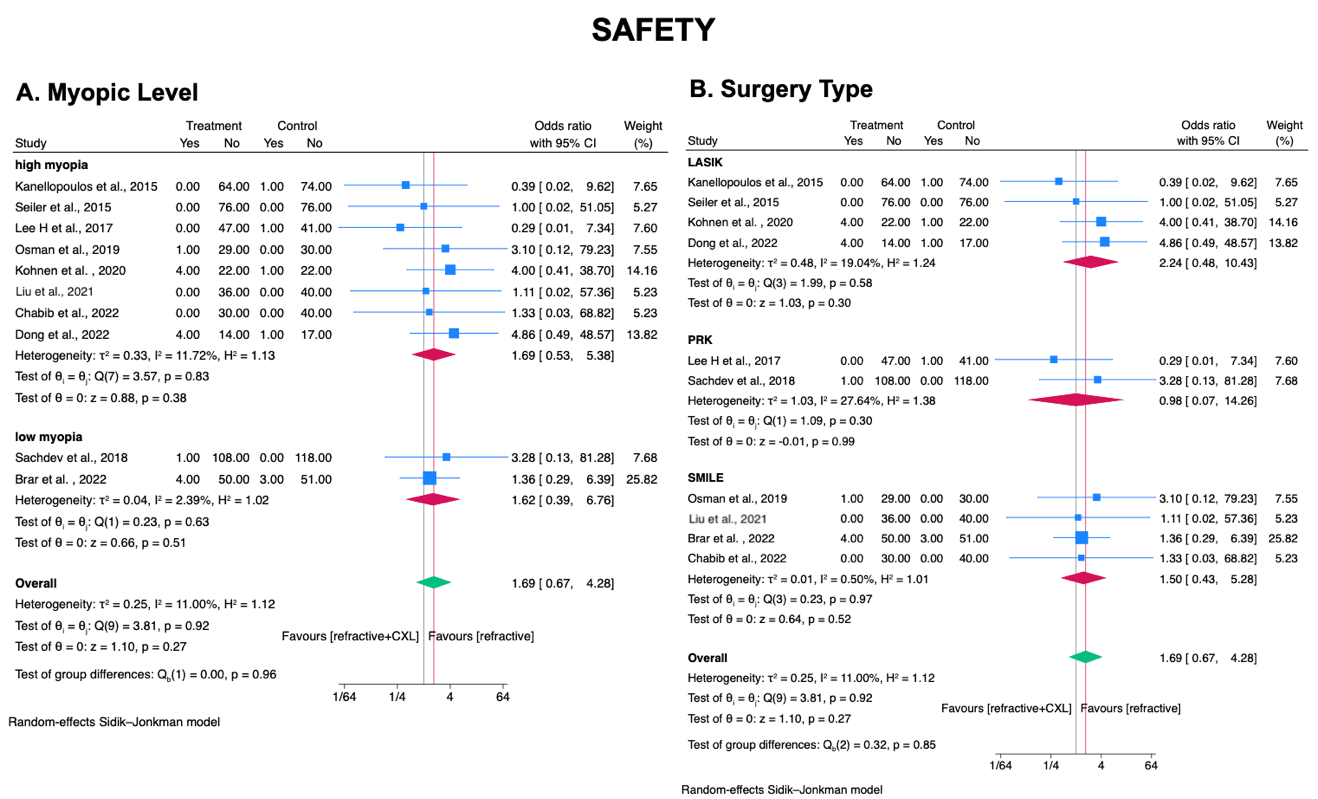


**eFig 11. Forest plot after stratification by (A) myopic level (B) laser refractive surgery type for comparison of prophylactic CXL plus laser refractive surgery with laser refractive surgery alone on safety in myopic patients at post-operative month twelve and month twenty-four. Safety defined as % of eyes with one or more lines of loss in CDVA.** CXL, cross-linking; SD, standard deviations.

Legend: The size of squares is proportional to the weight of each study. Horizontal lines indicate the 95% confidence intervals (CI) of mean difference estimate in each study; diamonds, the pooled estimate with 95% CI; N, the number of eyes at baseline; and SD, standard deviation.
