# Supplementary material for: Corneal stability comparison between prophylactic cross-linking with laser refractive surgery technique versus laser refractive surgery technique alone for myopia: a meta-analysis
Source: Graefes Arch Clin Exp Ophthalmol. 2025 Sep 11;263(11):3037–52. doi: 10.1007/s00417-025-06833-6 (PMC12675695; doi:10.1007/s00417-025-06833-6)

**Online resource 12. Sensitivity Analysis – Correlation Coefficients**

We repeated the meta-analysis using different values of correlation coefficient (0.5, 0.6, 0.7, and 0.8) to impute the SDs of the mean change in primary continuous outcome measures (K, MRSE, corneal thickness, ECD, UDVA and CDVA). Table S1 summarizes that in the study arm comparing prophylactic CXL with laser refractive surgery to laser refractive surgery alone, WMDs across the correlation coefficient of 0.5, 0.6, 0.7, and 0.8 were consistent.

eTable 12.1

| correlation coefficient | UDVA | CDVA | K | MRSE | Corneal thickness | ECD |
| --- | --- | --- | --- | --- | --- | --- |
| 0.5 | -0.02 [-0.05, 0.01] | 0.00 [-0.03, 0.03] | -0.02 [-0.46, 0.43] | 0.04［-0.01, 0.09］ | ﻿0.07［-12.47, 12.62］ | 4.99 [-57.06, 67.05] |
| 0.6 | -0.02 [-0.05, 0.01] | 0.00 [-0.03, 0.03] | -0.02 [-0.43, 0.39] | 0.04［-0.01, 0.09］ | ﻿0.07［-11.15, 11.30］ | 4.83 [-50.92, 60.59] |
| 0.7 | -0.02 [-0.05, 0.01] | 0.00 [-0.03, 0.03] | -0.02 [-0.40, 0.35] | 0.04［-0.01, 0.09］ | ﻿0.07［-9.64, 9.79］ | 4.58 [-44.06, 53.22] |
| 0.8 | -0.02 [-0.05, 0.01] | 0.00 [-0.03, 0.03] | -0.02 [-0.36, 0.31] | 0.04［-0.01, 0.08］ | ﻿0.07［-7.86, 8.01］ | 4.13 [-36.14, 44.41] |

In the study arm comparing prophylactic CXL with laser refractive surgery to laser refractive surgery alone, the WMDs of UDVA across the correlation coefficient of 0.5, 0.6, 0.7, and 0.8 were all -0.02 (95% CI, -0.05 to 0.01), the WMDs of CDVA across the correlation coefficient of 0.5, 0.6, 0.7, and 0.8 were all 0.00 (95% CI, -0.03 to 0.03), the WMDs of K across the correlation coefficient of 0.5, 0.6, 0.7, and 0.8 were all -0.02(95% CI, -0.43 to 0.39), the WMDs of MRSE across the correlation coefficient of 0.5, 0.6, 0.7, and 0.8 were all 0.04 (95% CI, -0.01 to 0.09), the WMDs of corneal thickness across the correlation coefficient of 0.5, 0.6, 0.7, and 0.8 were all 0.07 (95% CI, -11.15 to 11.30), and the WMDs of ECD across the correlation coefficient of 0.5, 0.6, 0.7, and 0.8 were all around 4.83 (95% CI, -50.92 to 60.59).

**Results of sensitivity analysis with different correlation coefficient (0.5, 0.6, 0.7, 0.8):**

**eFigure 12.1 UDVA**


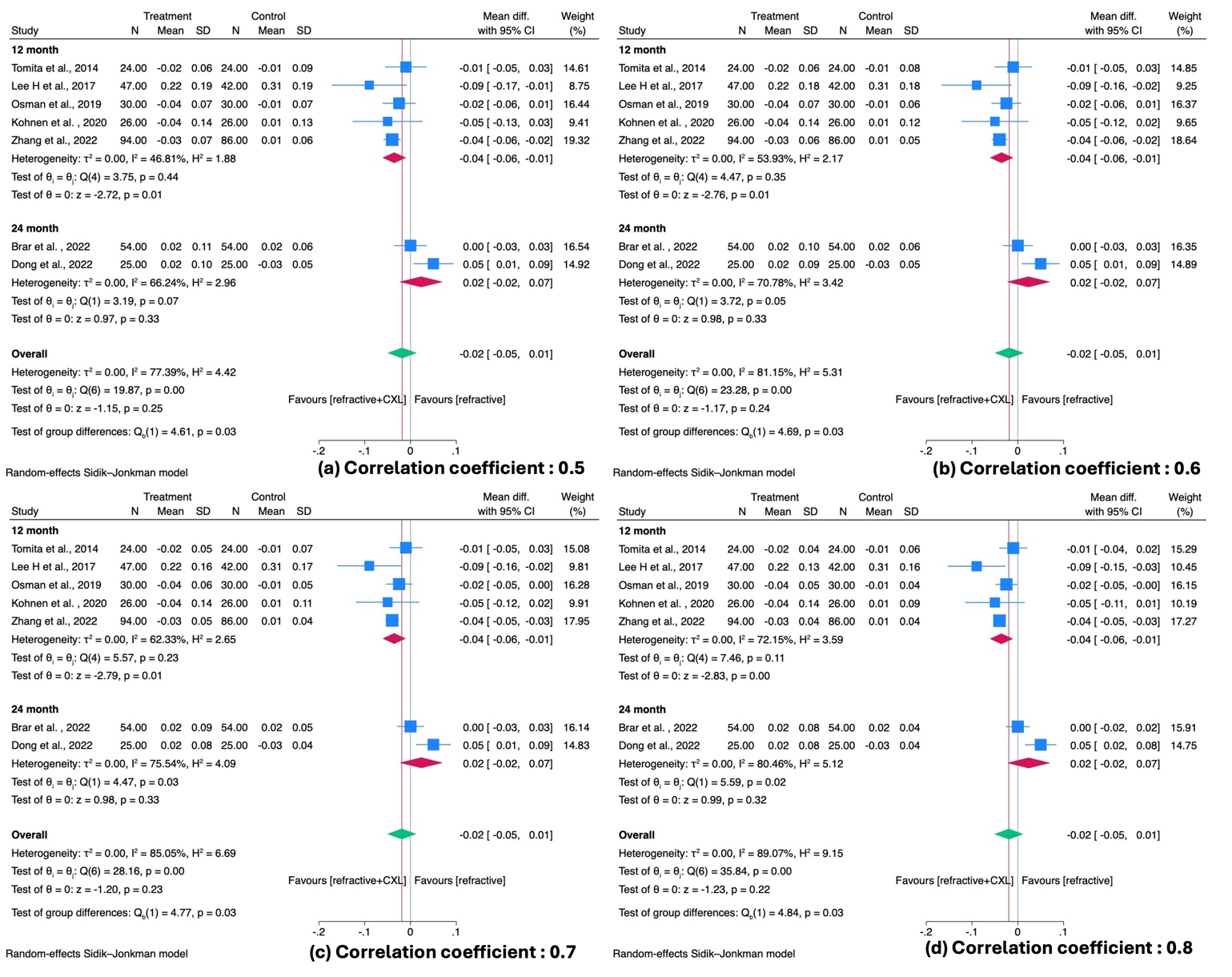


**eFigure 12.2 CDVA**


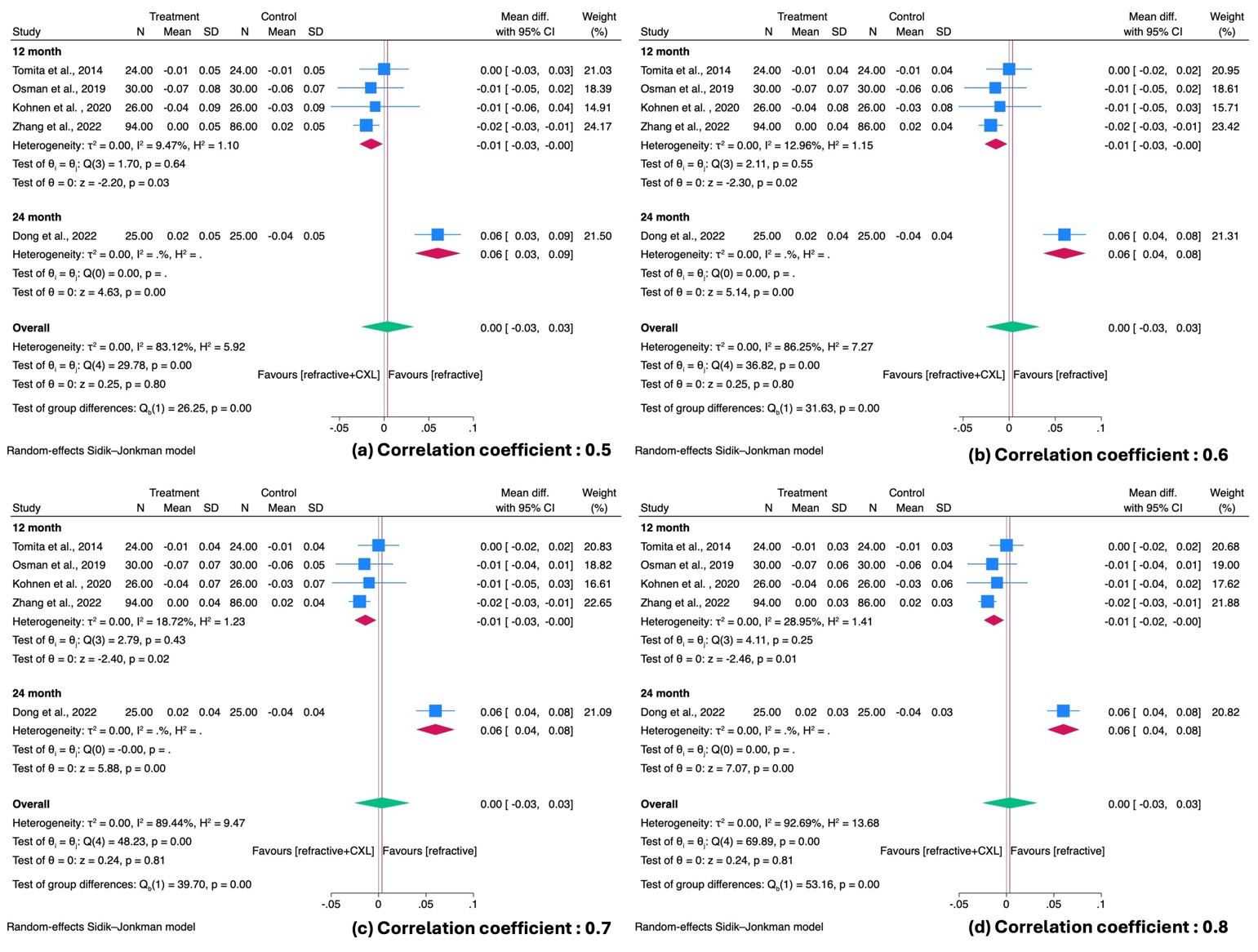


**eFigure 12.3 K**


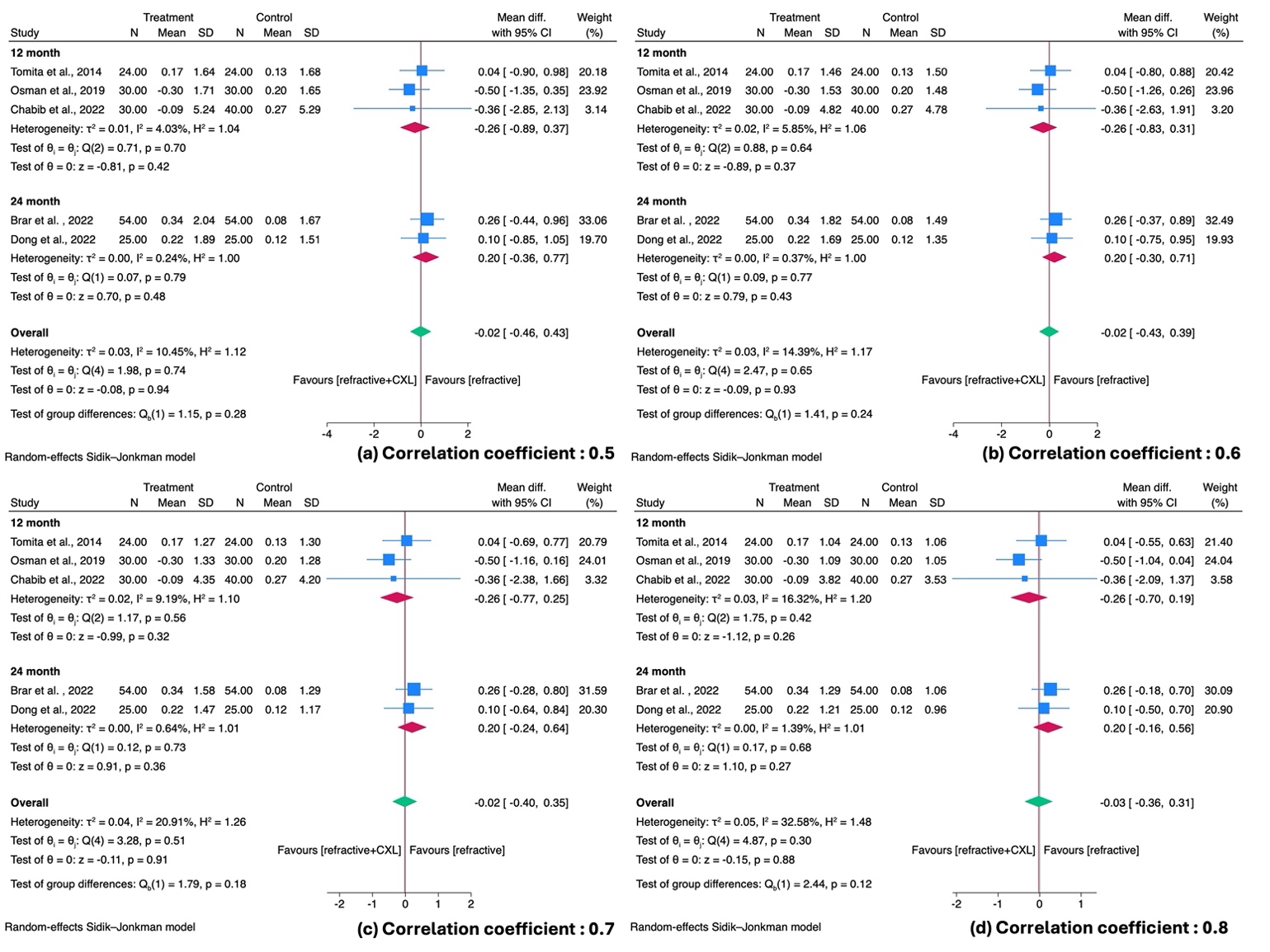


**eFigure 12.4 MRSE**


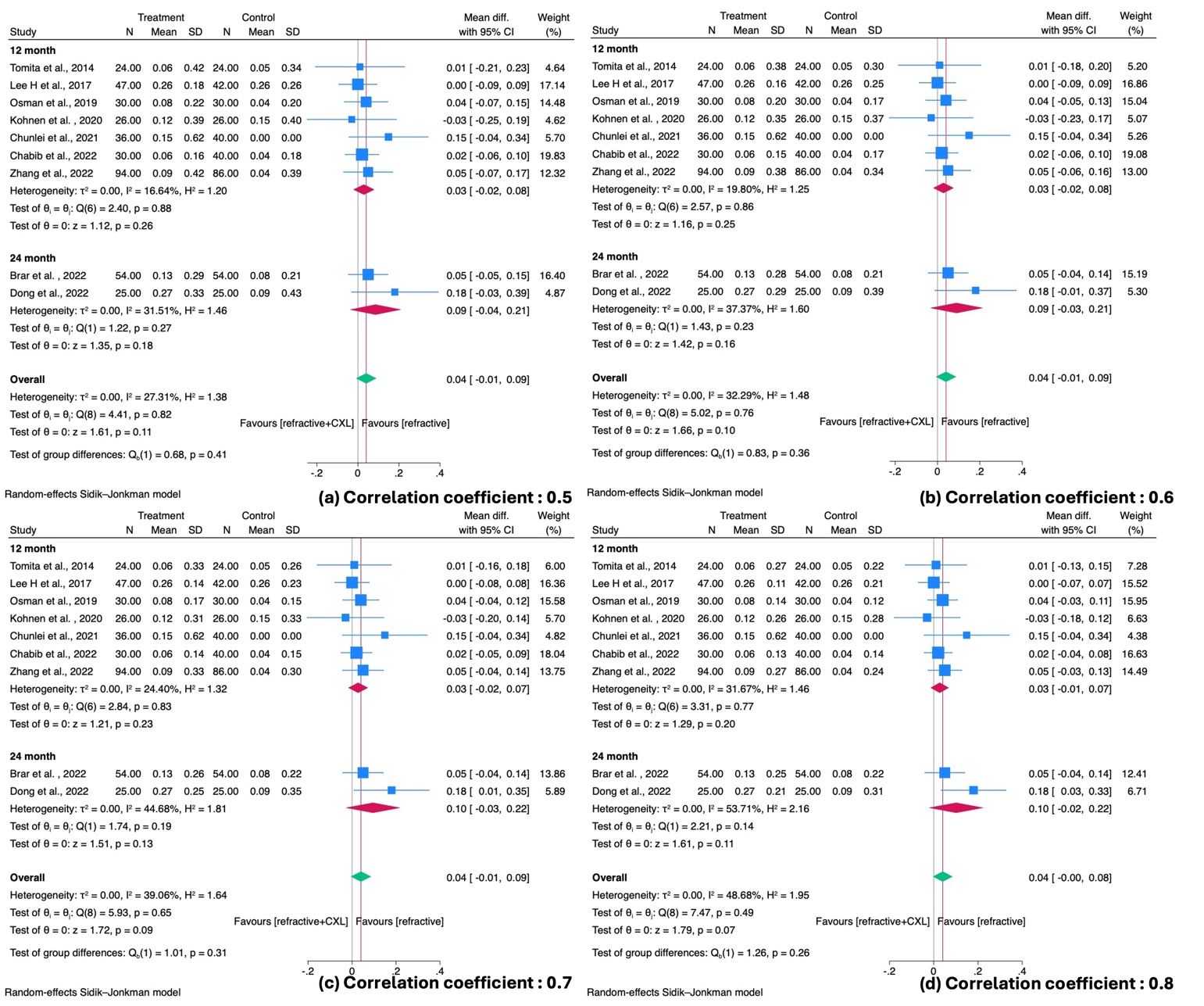


**eFigure 12.5. Corneal thickness**


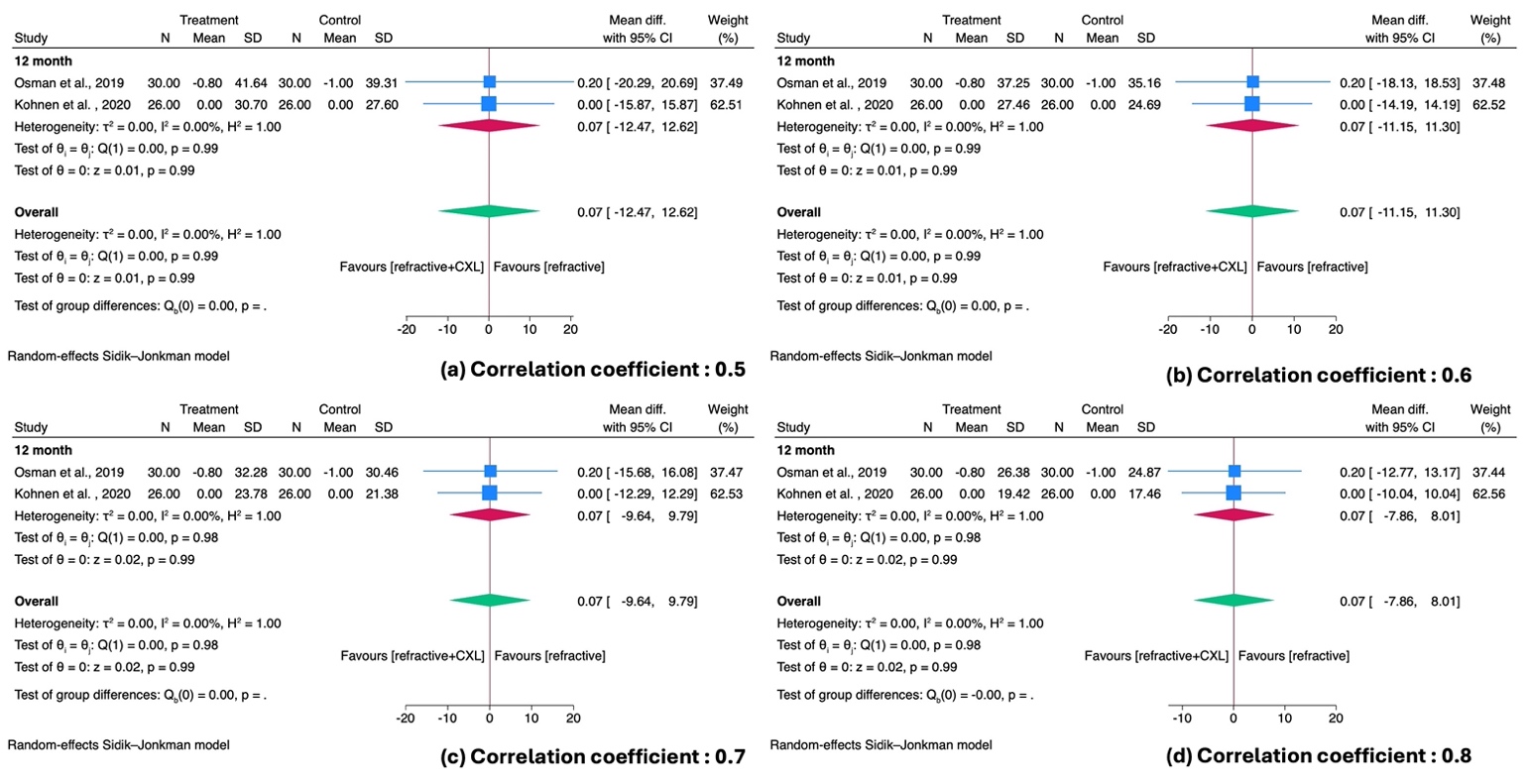


**eFigure 12.6 ECD**


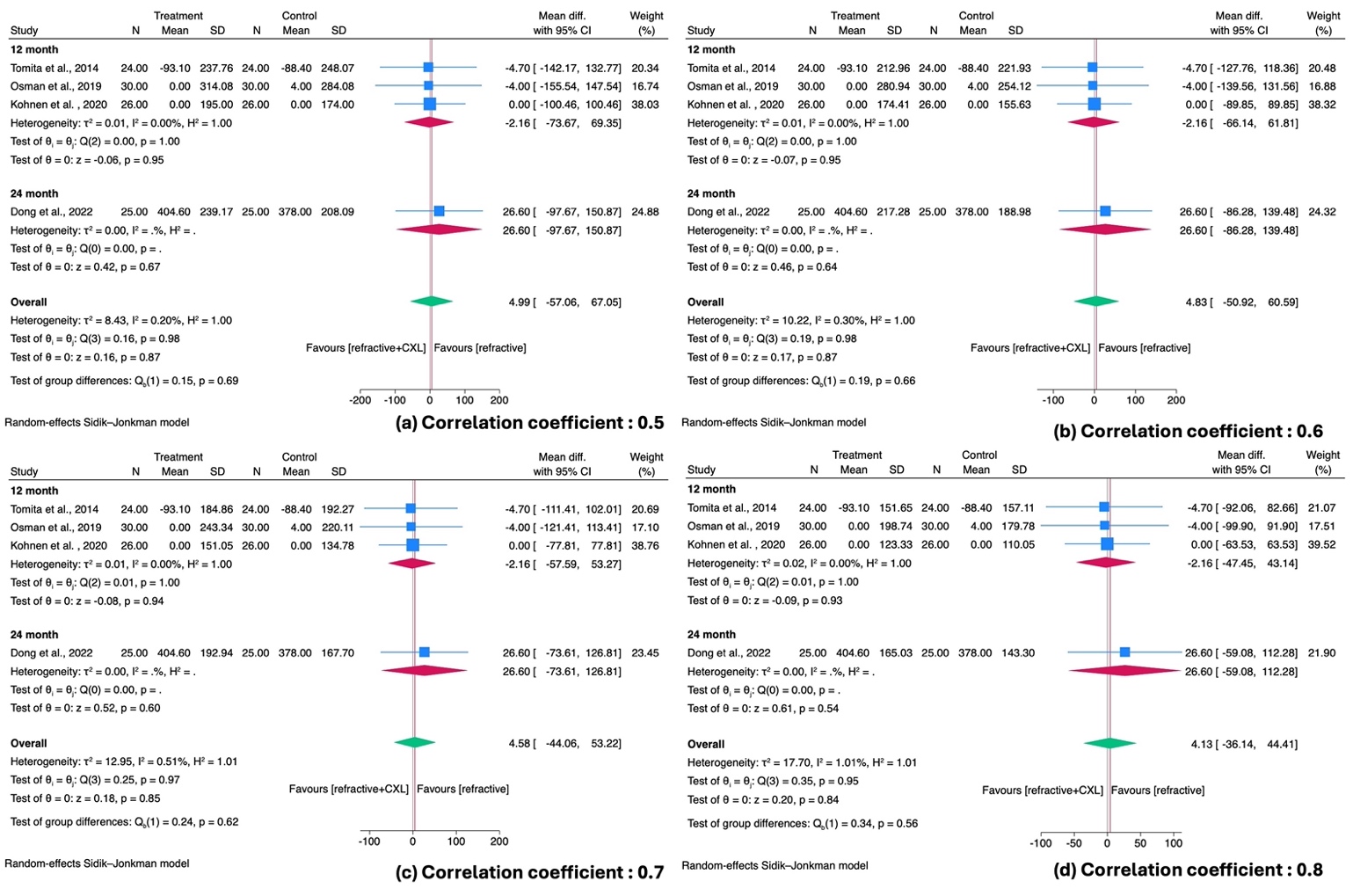

Supplement: Supplementary file 12 — Supplementary file12 (DOCX 2904 KB) [file 417_2025_6833_MOESM12_ESM.docx]
