# Supplementary material for: Corneal stability comparison between prophylactic cross-linking with laser refractive surgery technique versus laser refractive surgery technique alone for myopia: a meta-analysis
Source: Graefes Arch Clin Exp Ophthalmol. 2025 Sep 11;263(11):3037–52. doi: 10.1007/s00417-025-06833-6 (PMC12675695; doi:10.1007/s00417-025-06833-6)
Supplement: Supplementary file 13 — Supplementary file13 (DOCX 16184 KB) [file 417_2025_6833_MOESM13_ESM.docx]

**Online resource 13. Funnel Plot**

**eFigure 13.1 Funnel plot of studies included in the analysis of (A) UDVA and (B) CDVA stability.** UDVA, uncorrected distance visual acuity; CDVA, corrected distance visual acuity. Legend: Blue dots represent included studies.


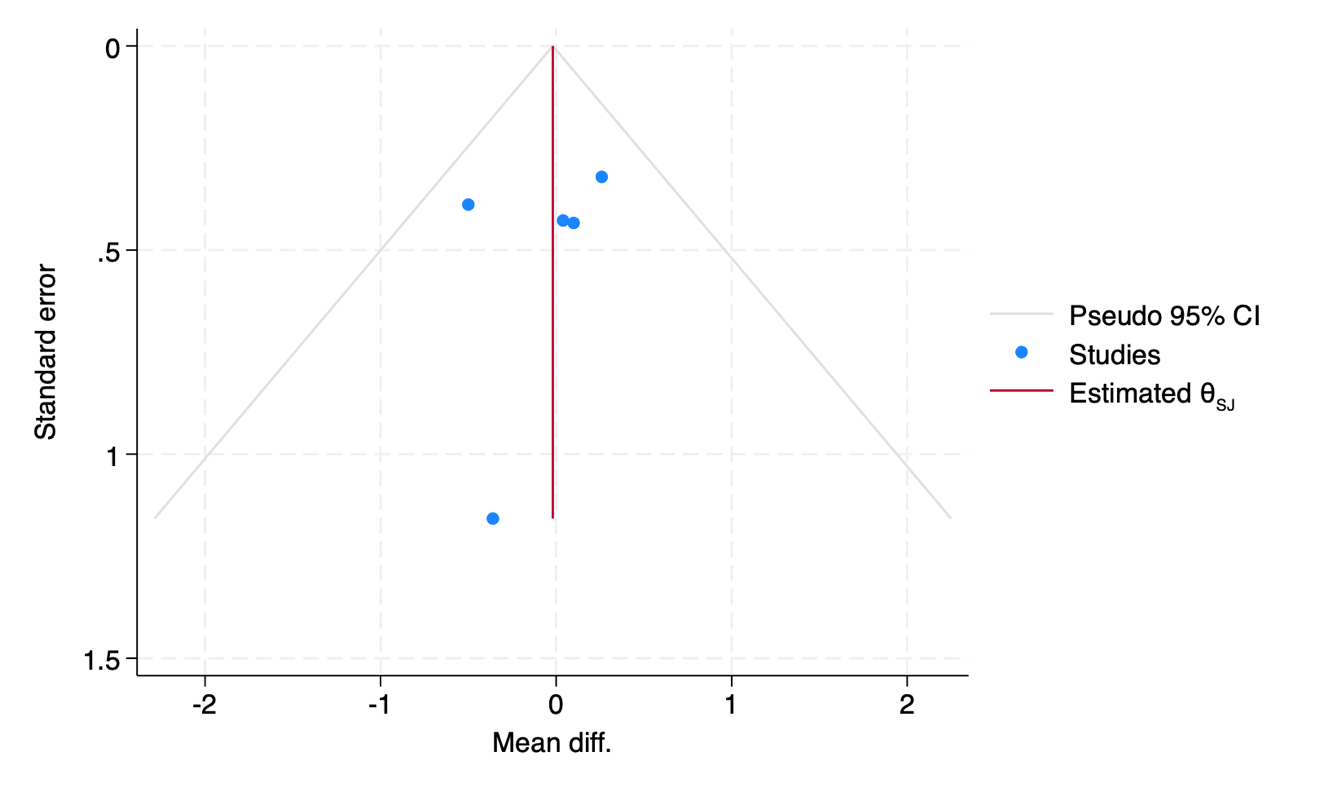


**eFigure 13.2 Funnel plot of studies included in the analysis of K stability.** K, keratometry. Legend: Blue dots represent included studies.


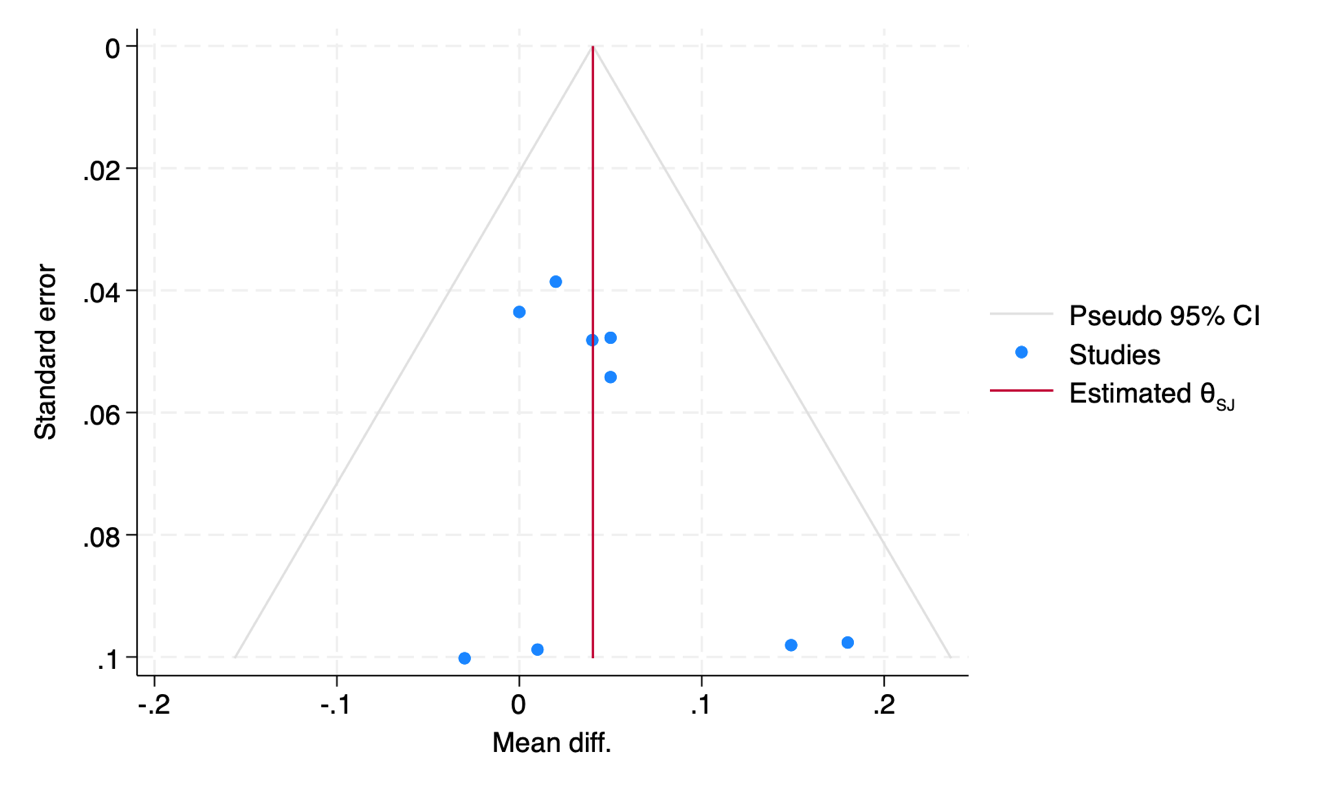


**eFigure 13.3 Funnel plot of studies included in the analysis of MRSE stability.** MRSE, manifest refraction spherical equivalent. Legend: Blue dots represent included studies.


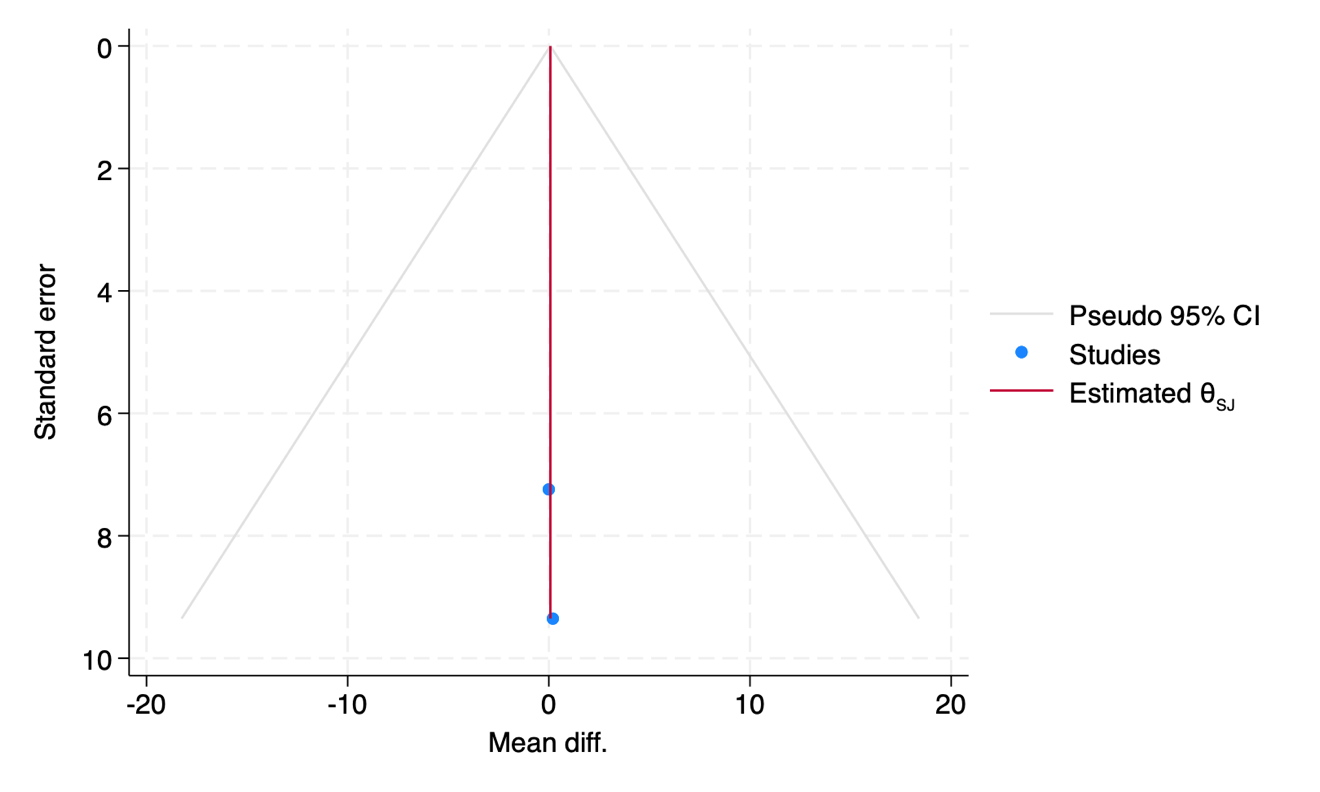


**eFigure 13.4 Funnel plot of studies included in the analysis of corneal thickness stability.** Legend: Blue dots represent included studies.


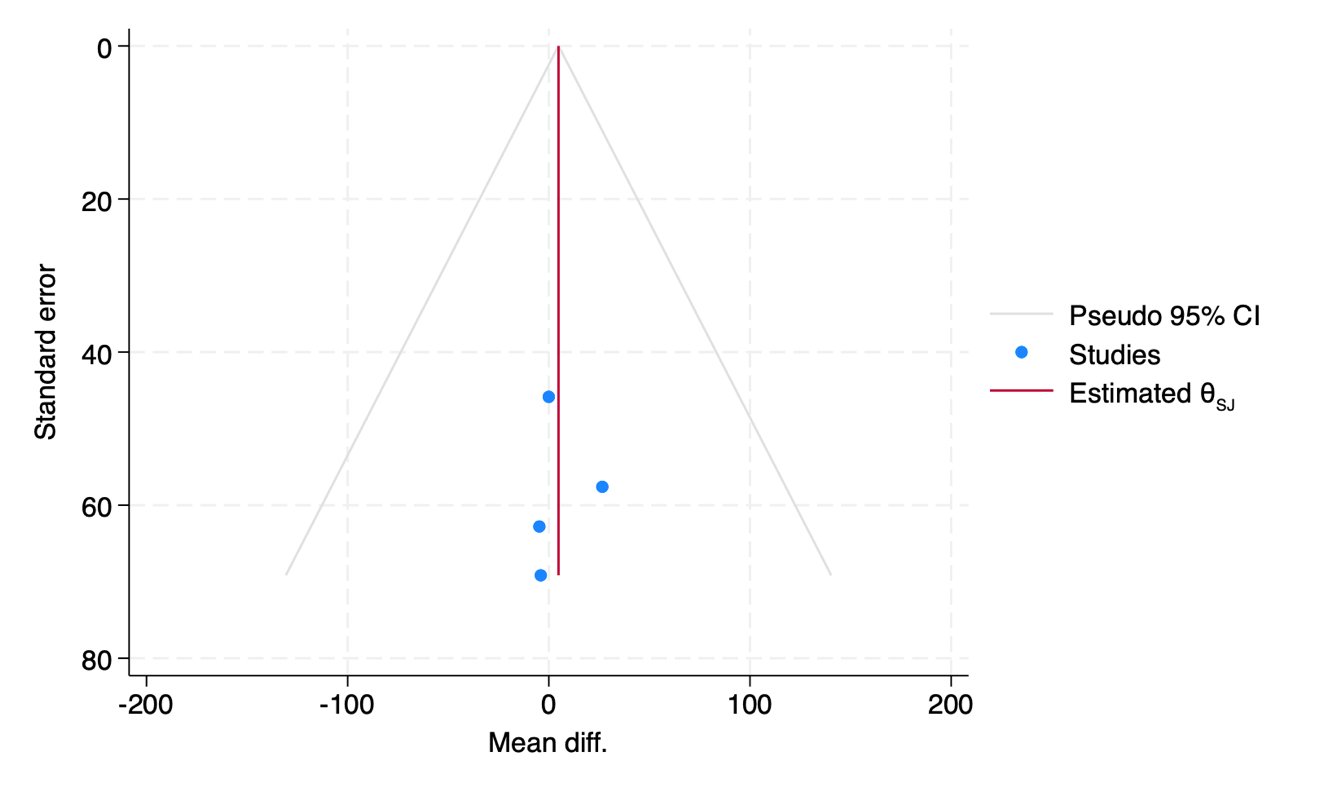


**eFigure 13.5 Funnel plot of studies included in the analysis of endothelial cell density stability**. Legend: Blue dots represent included studies.

**eFigure 13.6 Funnel plot of studies included in the analysis of efficacy.** Legend: Blue dots represent included studies.

**eFigure 13.7 Funnel plot of studies included in the analysis of predictability.** Legend: Blue dots represent included studies.

**
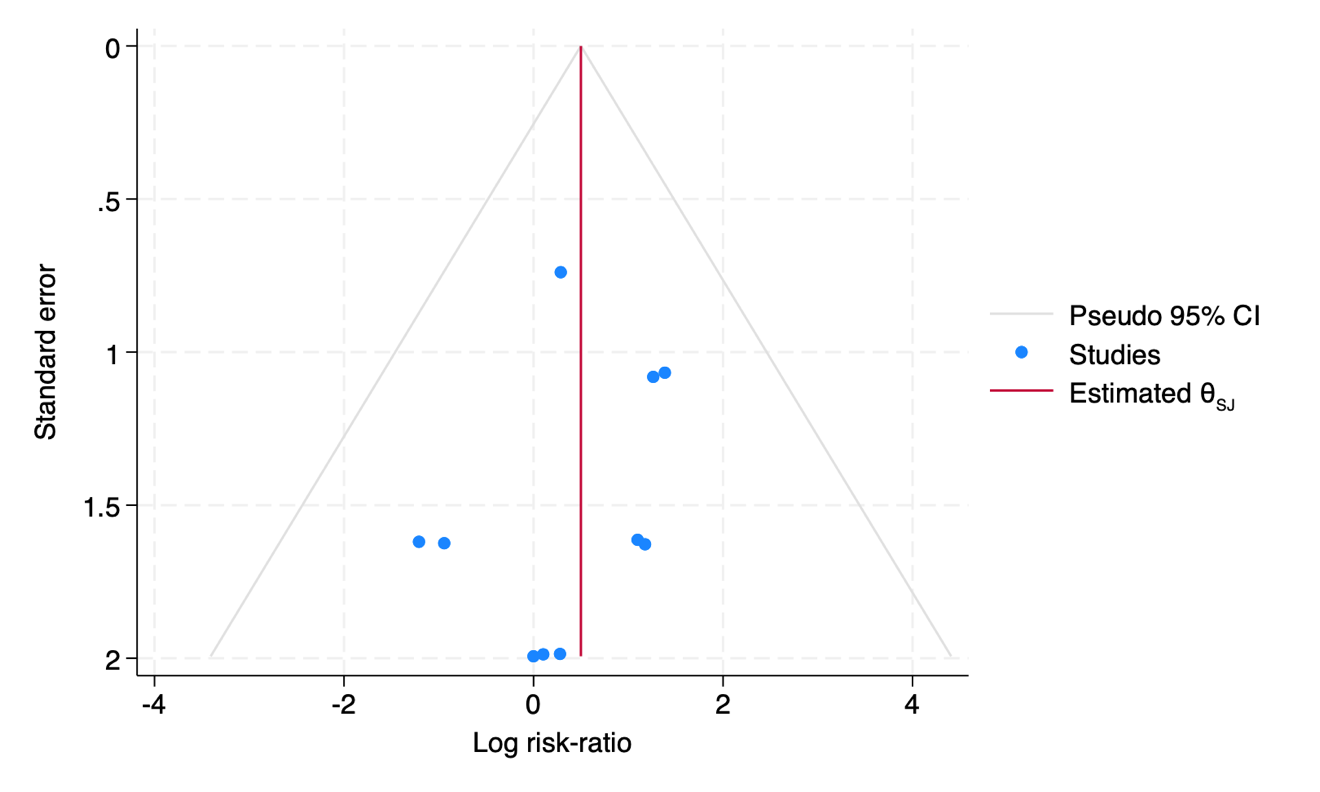
**

**eFigure 13.8 Funnel plot of studies included in the analysis of safety.** Legend: Blue dots represent included studies.
