# Supplementary material for: Corneal stability comparison between prophylactic cross-linking with laser refractive surgery technique versus laser refractive surgery technique alone for myopia: a meta-analysis
Source: Graefes Arch Clin Exp Ophthalmol. 2025 Sep 11;263(11):3037–52. doi: 10.1007/s00417-025-06833-6 (PMC12675695; doi:10.1007/s00417-025-06833-6)
Supplement: Supplementary file 14 — Supplementary file14 (DOCX 13 KB) [file 417_2025_6833_MOESM14_ESM.docx]

**Online resource 14. Reference**

1. Cochrane Handbook for Systematic Reviews of Interventions. Accessed May 10, 2020. /handbook

2. Ferris FL, Kassoff A, Bresnick GH, Bailey I. New Visual Acuity Charts for Clinical Research. *American Journal of Ophthalmology*. 1982;94(1):91-96. doi:10.1016/0002-9394(82)90197-0

3. Risk of bias tools. Accessed May 10, 2020. https://sites.google.com/site/riskofbiastool

4. Sterne JAC, Savović J, Page MJ, et al. RoB 2: a revised tool for assessing risk of bias in randomised trials. *BMJ*. Published online August 28, 2019:l4898. doi:10.1136/bmj.l4898
